# Physics-Informed Design and Bench/Phantom Validation of a Shaft-Compatible 13.56 MHz NFC System for Laparoscopic Colorectal Tumour Localisation

**DOI:** 10.3390/s26185759

**Published:** 2026-09-10

**Authors:** Bogdan Mocan, Mihaela Mocan, Mircea Fulea, Mircea Murar, Zsolt Mate, Adrian Calborean, Vasile V. Bintintan

**Affiliations:** 1 Department of Engineering Design and Robotics, Technical University of Cluj-Napoca, 400114 Cluj-Napoca, Romania; bogdan.mocan@muri.utcluj.ro (B.M.); mircea.fulea@staff.utcluj.ro (M.F.); mircea.murar@muri.utcluj.ro (M.M.); 2 Department of Internal Medicine, Iuliu Hațieganu University of Medicine and Pharmacy, 400012 Cluj-Napoca, Romania; 3 Department of Circuit Design, Tehnologistic, 407035 Cluj-Napoca, Romania; zsolt.mathe@tehnologistic.ro; 4 National Institute for Research and Development of Isotopic and Molecular Technologies, Donath Street, No 67-103, 400293 Cluj-Napoca, Romania; adrian.calborean@itim-cj.ro; 5 Department of Surgery, 1st Clinic of Surgery, Iuliu Hatieganu University of Medicine and Pharmacy, 400012 Cluj-Napoca, Romania; vasile.bintintan@umfcluj.ro

**Keywords:** RFID, NFC, 13.56 MHz, tumour localisation, laparoscopic surgery, colorectal cancer, ferrite antenna, physics-informed design, magneto-quasi-static sensing, biomedical instrumentation

## Abstract

Background/Objectives: Accurate intraoperative tumour localisation remains challenging in minimally invasive colorectal surgery because tactile palpation is lost and conventional markers can migrate or provide imprecise localisation. Building on a preceding tri-frequency study that identified 13.56 MHz as the preferred RFID band for the intended application, this work develops a shaft-compatible NFC antenna–reader platform and evaluates its electromagnetic behaviour from bench-top reference media to five-layer tissue-equivalent phantoms. Methods: A Ø3 × 25 mm Fair-Rite Material 67 ferrite-rod antenna was designed from material and geometric parameters using finite-rod demagnetisation, inductance, resonance, and field calculations, followed by FEM cross-validation and experimental characterisation. The primary dataset comprised 480 detection distance measurements (2 media × 4 tag angles × 30 repetitions × 2 encapsulation variants). Phantom testing added 1440 measurements at 22 °C and 600 measurements at 37 °C across three fabrication batches, with the 37 °C non-coaxial subset limited to one batch. Results: The fabricated antenna measured 16.9 µH versus a 17.4 µH analytical estimate (−2.9%), with loaded Q = 23. The coaxial detection range was 16.45 ± 0.29 mm in air and 16.26 ± 0.21 mm in saline; angle was the dominant determinant of range (partial η^2^ = 0.989). In the multi-layer phantom, detection was 100% at 0 and 10 mm perirectal fat thickness under coaxial alignment at 22 °C, whereas performance declined markedly with angular misalignment and no detections occurred at fat thicknesses ≥ 20 mm. Across detectable phantom configurations, FEM showed r^2^ = 0.994, RMSE = 0.81 mm, and mean bias +0.70 mm. Bare and resin-overcoated tags showed no statistically detectable range difference. Multi-tag discrimination reached 100% for up to three tags separated by ≥20 mm under coaxial alignment, but deteriorated with angular misalignment. Conclusions: The study demonstrates a physics-informed route from antenna miniaturisation to measured system performance, and defines the present operating envelope under controlled bench and tissue-equivalent phantom conditions. The electromagnetic measurements apply to the antenna–electronics subassembly; integrated-shaft, multi-prototype, multi-operator, ex vivo, and in vivo validation remain necessary before clinical performance can be determined.

## 1. Introduction

**Figure 1:**
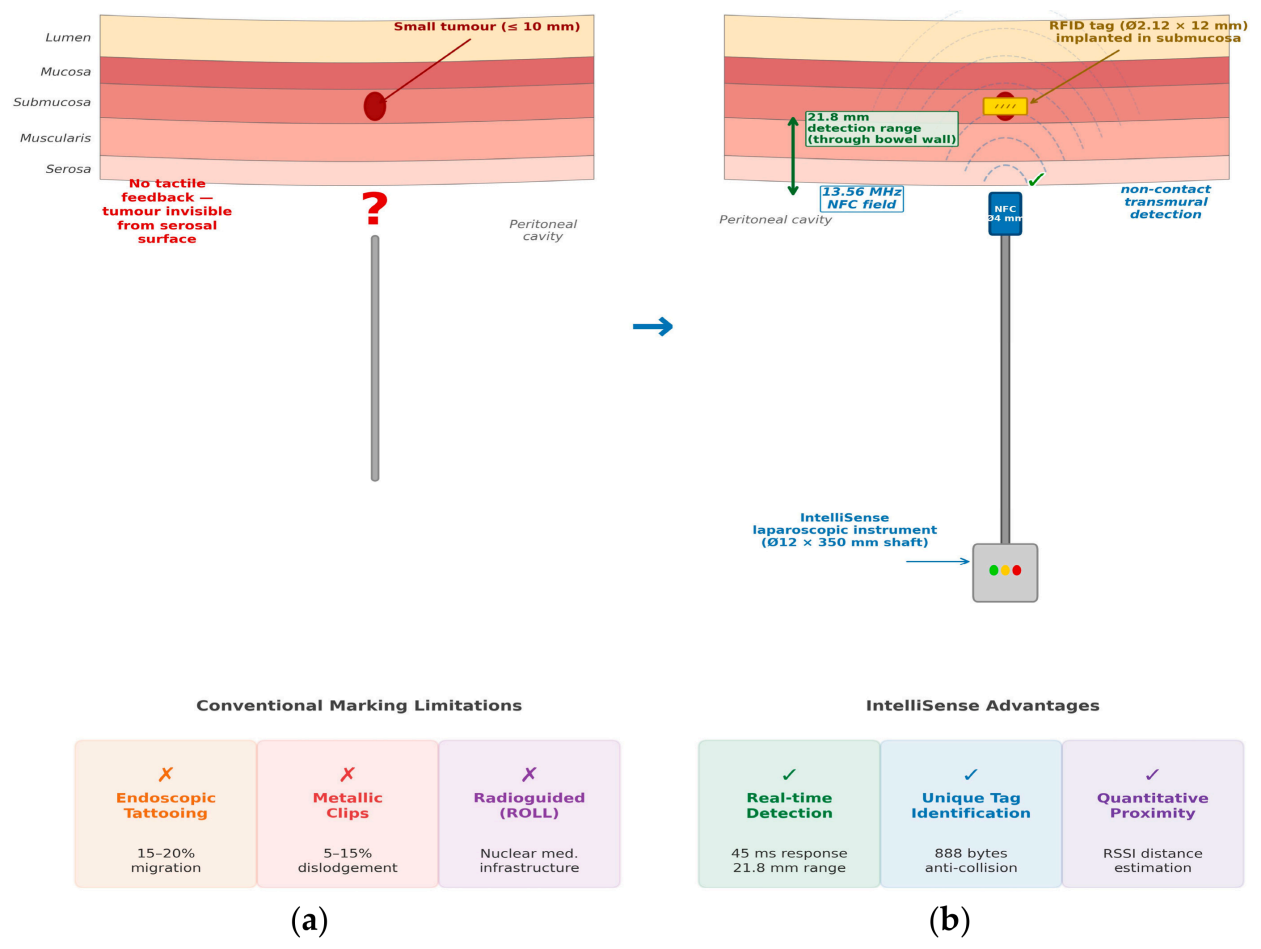
Clinical problem and proposed IntelliSense RFID-based transmural detection concept for laparoscopic colorectal tumour localisation. (**a**) Clinical challenge: loss of tactile feedback in laparoscopic surgery. (**b**) Proposed solution: 13.56 MHz NFC reader detecting an implanted transponder through the bowel wall. Colours are schematic and correspond to the labelled tissue/functional elements; blue dashed arcs in panel (**b**) represent the NFC magnetic-field coupling region, the green double arrow denotes detection range, and the coloured indicator dots represent instrument feedback.

Colorectal cancer is the third most common malignancy worldwide and the second leading cause of cancer-related death, with GLOBOCAN 2022 placing the annual burden at roughly 1.93 million new cases [1,2,3]. Minimally invasive surgery (MIS) has steadily displaced open resection, matching its oncological outcomes while easing recovery [4,5]. What MIS cannot restore, however, is touch; laparoscopic instruments remove the tactile feedback a surgeon would otherwise use to palpate tumour margins through the bowel wall—a capability open surgery takes for granted, and one that matters most for small, non-bulky, or post-neoadjuvant lesions that barely distort the serosal surface [6,7]. Figure 1 sets out this clinical problem alongside the proposed radio-frequency identification (RFID)-based solution, IntelliSense.

Standard practice still leans on preoperative endoscopic tattooing with India ink or carbon-based suspensions—a method whose drawbacks are well documented; markers migrate along tissue planes (in 15–20% of cases), diffuse circumferentially into imprecise localisation zones, and provoke inflammatory complications [8,9,10]. Metallic clips fare little better, with dislodgement rates of 5–15% [11], while radioguided occult lesion localisation depends on nuclear medicine infrastructure that many surgical centres simply do not have [12].

Wireless electromagnetic localisation is already established in other surgical domains. Radiofrequency localisation systems used in breast surgery demonstrate the feasibility of wireless marker retrieval [13,14,15,16], while inductive proximity sensors developed for gastrointestinal surgery can detect metallic markers through tissue [17,18,19]. More recently, RFID-integrated endoscopic clips have also been investigated for laparoscopic marking [20]. These approaches differ in carrier frequency, marker architecture, antenna dimensions, and clinical workflow. For colorectal use, the engineering challenge is therefore not the introduction of a new localisation principle, but the translation of RFID/NFC detection into a geometry compatible with laparoscopic access while retaining adequate range and unique marker identification.

Our preceding tri-frequency electromagnetic characterisation [21] compared 134 kHz, 13.56 MHz, and 868 MHz using glass-encapsulated transponders in air and physiological saline. That study selected 13.56 MHz as the most suitable compromise for the intended through-wall localisation task, but the HF reference antenna (Ø5 × 15 mm, 10-turn winding) was not compatible with the dimensional envelope targeted here. The present study therefore addresses the next engineering stage, namely, miniaturisation of the 13.56 MHz reader antenna, hardware characterisation of the as-built antenna–electronics subassembly, and the extension of detection testing from reference media to multi-layer tissue-equivalent phantoms.

Three contributions are evaluated. First, the antenna is redesigned as a Ø3 × 25 mm Material 67 ferrite-rod configuration whose effective permeability, inductance, resonance, and axial field are calculated from geometry and manufacturer material parameters. Second, the analytical field profile is cross-checked against FEM and measured antenna parameters, while the mapping from field strength to tag detection is treated explicitly as an empirically anchored system-level model rather than as a fully independent ab initio prediction. Third, the experimental programme quantifies the detection envelope as a function of propagation medium, tag angle, encapsulation, multi-tag conditions, and layered phantom geometry. Model-based localisation projections are retained only as a prospective methodology in the Supplementary Materials, and are not used as evidence of measured localisation accuracy.

The study encompasses bench-top characterisation of the antenna–electronics subassembly in air and 0.9% NaCl, followed by validation in five-layer tissue-equivalent phantoms. These results support the TRL 4 laboratory validation of the sensing principle and define the conditions under which the present prototype operates reliably. Electromagnetic verification of the fully integrated shaft, multi-prototype and multi-operator reproducibility, tag migration/rotation, ex vivo tissue performance, and subsequent in vivo evaluation are addressed in subsequent validation stages.

## 2. Materials and Methods

### 2.1. Requirements Engineering Framework

The IntelliSense system was developed within a requirements-driven design framework informed by ISO 13485:2016 quality-management principles and IEC 62366-1:2015 usability-engineering guidance [22,23]. Stakeholder analysis covered surgical users, endoscopy users, patients, hospital procurement, regulatory authorities, biomedical engineering, and sterilisation services. The requirements elicitation comprised twelve semi-structured clinical interviews and twelve operating-room procedural observations, yielding sixteen user needs. The engineering subset was translated into quantified design inputs, while requirements intended for later-stage verification are retained in the traceability matrix as open programme-level items (Supplementary Table S1).

Nine requirements were classified as Must-Have acceptance criteria for the development programme; Table 1 lists the criteria relevant to the present laboratory validation phase together with their current evidential status. The terminology distinguishes (i) the experimentally verified performance of the tested antenna–electronics subassembly, (ii) the analytical/model-based design quantities cross-checked against measurement, and (iii) the regulatory or integrated-device activities that remain pending. Accordingly, the ≥15 mm detection criterion is evaluated under the predefined coaxial reference medium condition, while orientation- and tissue-dependent performance are assessed separately in Section 3.8.

The 15 mm range target was derived as an engineering design objective from the anticipated bowel wall plus local tissue path. It is used here as a coaxial reference condition benchmark rather than a universal acceptance criterion across all tag orientations and tissue geometries. The multi-layer phantom results in Section 3.8 demonstrate that angular misalignment and perirectal fat thickness substantially narrow the usable envelope. The operating frequency of 13.56 MHz was fixed a priori by the tri-frequency selection study [21].

### 2.2. System Architecture

**Figure 2:**
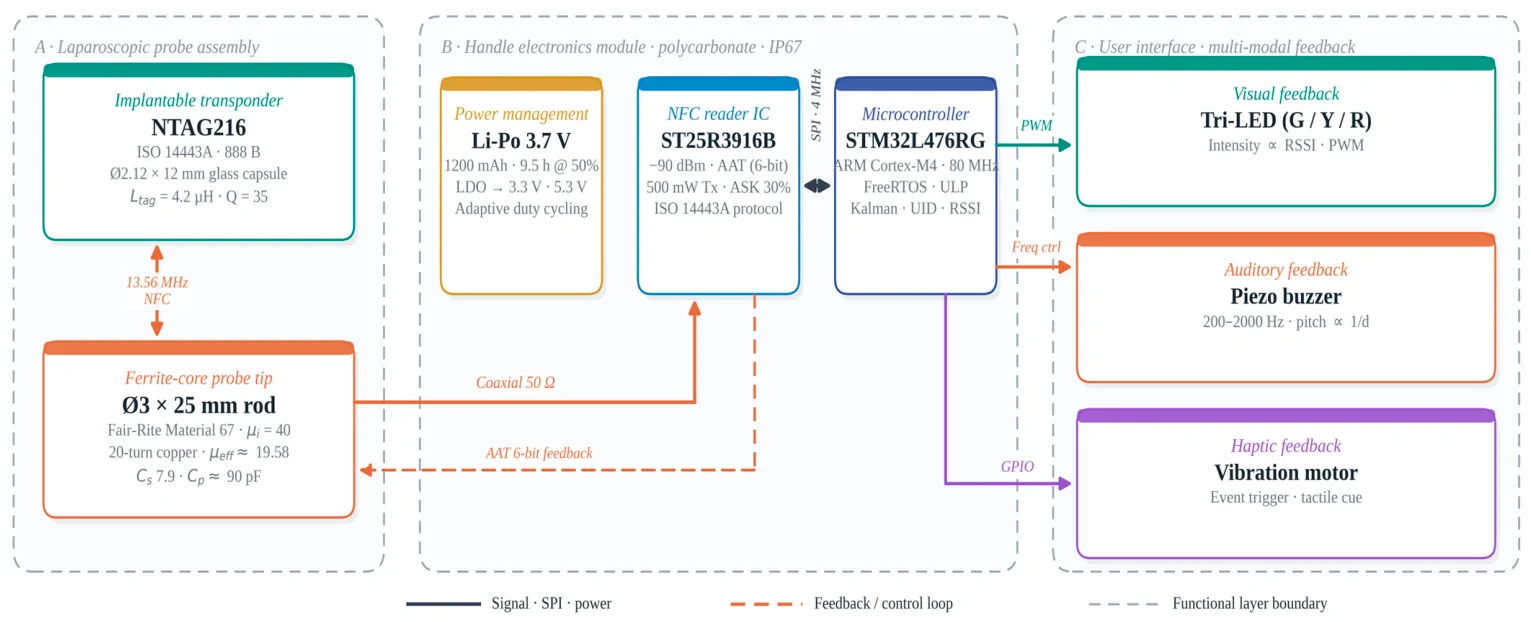
System block diagram of the IntelliSense laparoscopic instrument, showing the implantable NTAG216 glass-encapsulated transponder, the Ø3 × 25 mm Material 67 ferrite-rod antenna at the distal probe tip, the ST25R3916B NFC transceiver with automatic antenna tuning, the ARM Cortex-M4 host microcontroller, the multi-modal user-interface block, and the Li-Po battery power management subsystem [26].

The IntelliSense system architecture comprises four principal subsystems: (i) an implantable glass-encapsulated NTAG216 NFC transponder (NXP Semiconductors, Eindhoven, The Netherlands) anchored near the tumour site during preoperative endoscopy; (ii) a ferrite-core reader antenna of Ø3 × 25 mm using Fair-Rite Material 67 (Fair-Rite Products Corporation, Wallkill, NY, USA), optimised for the laparoscopic shaft envelope; (iii) an NFC reader electronics module incorporating the ST25R3916B transceiver and STM32L476RG ARM Cortex-M4 host microcontroller (STMicroelectronics, Geneva, Switzerland), together with automatic antenna tuning; and (iv) a multi-modal user interface delivering visual, auditory, and haptic detection feedback to the surgeon. Figure 2 presents the complete system block diagram.

#### Hierarchical Control Architecture

Beyond the functional block diagram in Figure 2, the IntelliSense system implements a hierarchical control architecture organised in four functional layers governing real-time detection, power management, antenna tuning, and safety monitoring (Figure S1). The primary detection loop constitutes the critical real-time signal path—upon transmission of a 13.56 MHz interrogation pulse by the ST25R3916B reader IC, the electromagnetic field couples inductively with the implanted NTAG216 transponder through the ferrite-core antenna; the backscattered ISO 14443-A response is demodulated and the RSSI plus 7-byte UID extracted, after which the ARM Cortex-M4 host microcontroller (STM32L476RG, 80 MHz) executes the firmware decision logic. The complete detection loop closes within 45 ± 8 ms (Section 3.7), enabling real-time proximity feedback at update rates exceeding 22 Hz—well within the human perceptual threshold for continuous guidance during probe manipulation.

The power-management loop implements adaptive duty cycling to balance detection responsiveness against battery conservation, as follows: in search mode (no transponder detected), the duty cycle reduces to 30% with 100 ms polling intervals, while upon initial detection the duty cycle increases to 100% continuous scanning for precise proximity tracking. This adaptive strategy yields 9.5 ± 0.3 h of battery autonomy at the nominal 50% duty cycle from the 1200 mAh Li-Po cell (Section 3.7), substantially exceeding the 4-h Must-Have specification.

The antenna-tuning loop uses the ST25R3916B antenna-tuning control outputs to drive an external variable-capacitance network, compensating detuning of the matched antenna circuit. Resonance monitoring is based on the reader phase/amplitude measurement functions, with the tuning algorithm implemented in the host firmware. The safety monitoring functions implemented in the prototype are limited to UID verification, fault detection, and RF shutdown. Formal electromagnetic compatibility and human-exposure compliance were not established in the present study, and remain pre-clinical qualification activities.

The 13.56 MHz operating frequency, fixed by the tri-frequency comparative analysis of Mocan et al. (preceding study), operates in the magneto-quasi-static regime with electromagnetic wavelength λ ≈ 22.1 m, vastly exceeding all relevant system dimensions (L/λ ≈ 10^−3^ at the maximum measured detection distance of 16.45 mm). The fundamental coupling mechanism is therefore inductive near-field, with magnetic flux density decaying with r^−3^ in the dipole far field of the antenna and the energy transferred to the transponder governed by mutual inductance. The selection rationale and tri-band performance benchmarks have been comprehensively presented in the preceding study and are not repeated here; the present work focuses on the design-engineering challenge of translating the bench-top HF reference antenna into a shaft-compatible configuration.

The geometric design progression from the [21] reference entailed two coupled modifications. The reader antenna diameter was reduced from Ø5 mm to Ø3 mm to meet the shaft-compatible design envelope, while rod length was increased from 15 mm to 25 mm to increase aspect ratio and reduce demagnetisation. For L/D = 8.33, the prolate-ellipsoid approximation gives N_z ≈ 0.027 (Section 2.4). Material 67 was selected as a NiZn ferrite suitable for HF operation. The resulting analytical design gives μ_eff = 19.58, L_ant = 17.4 µH, and C_res = 7.9 pF. Loss and unloaded-Q calculations are treated as idealised analytical estimates; the experimentally relevant loaded quality factor is measured directly in Section 3.1.

### 2.3. RFID Transponder Selection and Characterisation

The implantable transponder constitutes the marker intended for preoperative endoscopic placement near the lesion. Selection criteria included hermetic encapsulation, dimensional compatibility with a dedicated endoscopic delivery device of sufficient internal diameter, resonance near 13.56 MHz, and the availability of a unique identifier supporting multi-marker protocols. The delivery mechanics, long-term anchoring, migration, and rotation of the implanted tag were not validated in the present bench/phantom study, and are addressed as translational limitations in Section 4.6.

The reference transponder uses an NTAG216 IC (NXP Semiconductors, Eindhoven, The Netherlands) incorporated into a borosilicate-glass transponder assembly (Ø2.12 × 12 mm), providing 888 bytes of user memory, NFC Forum Type 2/ISO/IEC 14443-A support, and nominal IC input capacitance of 50 pF [27]. Resonance is established by the transponder antenna/coil and associated tuning capacitance; it is not an on-chip microcoil property of the NTAG216 IC. The glass capsule provides hermetic encapsulation, while device-level biological evaluation of the complete implanted marker remains a planned regulatory activity.

Two encapsulation variants of the NTAG216 glass tag were tested in the present work—a bare glass capsule (denoted *glass_2 mm_bare*) and a resin-overcoated variant (denoted *glass_2 mm_resin*) representing a candidate manufacturing process for enhanced mechanical robustness. The resin variant comprises a thin epoxy overlay (~150 µm) applied for surface protection during deployment through the injector. Both variants were characterised independently across the full 8-condition experimental matrix to verify that the additional dielectric overlay does not degrade electromagnetic coupling performance—a regulatory-relevant finding addressed quantitatively in Section 3.5.

Transponder-side parameters were characterised by vector network analysis on the bare-glass variant (L_tag = 4.2 µH, Q_tag = 35, measured resonance approximately 13.52 MHz in the tested fixture). This measured passive resonance should not be interpreted as evidence of compliance with the reader-carrier frequency tolerance. The activation field of the specific glass-encapsulated transponder was not independently measured in this study. Accordingly, H_ref = 3.0 A/m is retained only as a literature-informed reference value based on small-aperture NFC/PICC field-strength ranges [28], not as a certified activation threshold for the exact tag used here. The system-level field-to-detection mapping in Section 3.4 is therefore explicitly described as empirically anchored.

### 2.4. Ferrite-Core Reader Antenna: Physics-Informed Electromagnetic Design

The ferrite-core antenna is the principal determinant of detection range and the main engineering constraint in translating the larger reference antenna of [21] into a shaft-compatible geometry. The analytical sequence comprises effective permeability under demagnetisation (Section 2.4.1), inductance and resonance (Section 2.4.2), an idealised conductor/magnetic-loss estimate (Section 2.4.3), equivalent magnetic moment and axial-field profile (Section 2.4.4), and reader-side matching (Section 2.4.5). Quantities derived solely from geometry/material data are distinguished from the fitted or empirically anchored quantities used later to relate field strength to detection.

#### 2.4.1. Effective Permeability of the Finite Ferrite Rod

The antenna core is a cylindrical NiZn ferrite rod (Fair-Rite Material 67, part 3067990871), Ø3 × 25 mm, with aspect ratio L/D = 8.33. Material 67 was selected for its HF-use characteristics and moderate intrinsic permeability (μ_i ≈ 40) [29]. The 25 mm length increases aspect ratio within the available housing length and thereby limits demagnetisation relative to a shorter rod. Frequency-dependent loss parameters and winding parasitics are treated as uncertainty sources rather than as exact manufacturer constants in the interpretation of the unloaded-Q calculation.

For a finite cylindrical rod, the magnetic field inside the core is reduced from the closed-circuit toroidal value by the demagnetising field generated at the rod ends. The equivalent prolate-ellipsoid approximation [30] provides the axial demagnetising factor as

(1) $$N_{z}=\frac{1-e^{2}}{e^{3}}\left[\frac{1}{2}ln\frac{1+e}{1-e}-e\right],\;\mathrm{with}\;e=\sqrt{1-\frac{1}{{\left(L/D\right)}^{2}}}$$

with eccentricity e and aspect ratio L/D. For the present geometry (L/D = 8.33), Equation (1) yields e = 0.993 and N_z = 0.027. The approximation is recognised as accurate to better than 5% for cylindrical rods with L/D > 5 [30], which the present design comfortably satisfies. The effective permeability of the ferrite-rod antenna is then

(2) $$\mu_{eff}=\frac{\mu_{i}}{1+N_{z}\left(\mu_{i}-1\right)}=\frac{40}{1+0.0268\times 39}\approx 19.58$$

This value represents a 51% reduction from the intrinsic material permeability μ_i = 40 due to the finite-length demagnetisation, but provides 7.8× the flux concentration of an air-core coil of identical winding geometry. By comparison, the bench-top reference geometry of [21] (L/D = 3, μ_i = 125) yielded μ_eff ≈ 10.7—the present aspect ratio strategy therefore recovers approximately 83% of the bench-top reference effective permeability while operating with a 32% smaller intrinsic-permeability material, an outcome consistent with the design principle that a high aspect ratio compensates for moderate intrinsic permeability in finite-rod antennas operating at HF.

#### 2.4.2. Inductance and Resonance Tuning

The antenna winding comprises 20 turns of single-strand 0.2 mm enamelled copper wire arranged as a tightly packed helix of axial length l_c = 4.0 mm centred on the ferrite rod. Tight winding (coil length < rod length) is essential because the closed-form inductance expression for a solenoid embedded in a longer ferrite rod assumes that the winding is concentrated at the rod centre, where the demagnetisation-reduced effective permeability of Equation (2) is fully expressed; off-centre or distributed windings would display a position-dependent effective permeability and require numerical integration. The cross-sectional area of the ferrite core is

(3) $$A_{core}=\pi {\left(\frac{D}{2}\right)}^{2}=\pi {\left(1.5\times 10^{-3}\right)}^{2}=7.07\times 10^{-6}\;m^{2}$$

The antenna inductance follows the solenoid-with-core formula,

(4) $$L_{ant}=\frac{\mu_{0}\mu_{eff}N^{2}A_{core}}{l_{c}}=\frac{\left(4\pi \times 10^{-7}\right)\times 19.58\times 400\times \left(7.07\times 10^{-6}\right)}{4.0\times 10^{-3}}\approx 17.4\;\mu H$$

The capacitance required for resonance at the operating frequency f_0_ = 13.56 MHz is

(5) $$C_{res}=\frac{1}{\omega_{0}^{2}L_{ant}}=\frac{1}{{\left(2\pi \times 13.56\times 10^{6}\right)}^{2}\times 17.4\times 10^{-6}}\approx 7.9\;pF$$

This value is conveniently realised with a single C0G/NP0 ceramic capacitor of 8.2 pF E12 series value, with the residual frequency offset compensated by the automatic antenna-tuning mechanism described in Section 2.6.

#### 2.4.3. AC Resistance, Magnetic Losses, and Quality Factor

An idealised loss estimate was obtained from the calculated AC winding resistance and a ferrite-loss term (derivation in Supplementary Section S11). For the 0.2 mm copper wire at 13.56 MHz, the Howe round-conductor approximation [31] gives an AC/DC ratio of approximately 3.12 and R_AC ≈ 343 mΩ. Because proximity effect, self-capacitance, interconnection loss, and the frequency dependence of the complex ferrite permeability are not fully represented by the closed-form model, the resulting loss resistance is used only to establish an analytical upper bound on unloaded Q.

(6) $$R_{tot}=R_{AC}+R_{ferrite}\approx 343+58\approx 401\;m\Omega$$

The corresponding idealised value Q_unloaded ≈ 3700 is therefore not reported as a measured property of the fabricated antenna and should not be compared directly with practical reader-antenna Q values. Operational performance is instead characterised by the measured loaded quality factor Q_loaded = 23 (Section 3.1), which includes the matching network and reader loading required for NFC operation. The detailed analytical loss calculation is retained in Supplementary Section S11 as a design estimate with its limitations stated explicitly.

#### 2.4.4. Equivalent Magnetic Moment and Axial Field Profile

The equivalent magnetic moment is used to construct the analytical axial field profile. Under the nominal excitation used in the model, m = *N* · I · μ_eff · A_core, giving m = 0.277 × 10^−3^ A·m^2^ for *N* = 20, μ_eff = 19.58 and the assumed nominal antenna current. The current value is a modelling input rather than an independently calibrated field quantity. The axial magnetic field intensity is approximated by the finite-rod expression

(7) $$H\left(r\right)\approx \frac{m}{2\pi \;{\left(r^{2}+{\left(L/2\right)}^{2}\right)}^{3/2}}$$

where r is the axial distance measured from the centre of the rod. The presence of the (L/2)^2^ geometric correction term distinguishes the finite-rod expression from the point-dipole approximation H_dipole(r) = m/(2π r^3^); for the present geometry with L/2 = 12.5 mm, this correction accounts for ~38% of the denominator at r = 16 mm and is therefore essential for accurate field prediction. The angular dependence of the coupling at non-coaxial transponder orientations follows

(8) $$H_{coupled}\left(r,\theta \right)=H\left(r\right)cos\;\theta +H_{⊥}\left(r\right)sin\;\theta \;\eta_{⊥}$$

where η_⟂ is an empirical transverse-coupling coefficient introduced to account for non-axial field coupling at large tag angles. Because η_⟂ is obtained from experimental anchoring, Equation (8) is a hybrid physics-informed/empirical detection model rather than a fully independent first principles prediction. Full derivations and the transverse field representation are provided in Supplementary Section S12.

#### 2.4.5. Matching Network and Reader Coupling

The antenna matching network transforms the high-reactance ferrite coil impedance to the loading condition required by the ST25R3916B antenna driver [32]. The 50 Ω quantity used in VNA characterisation is an instrumentation reference impedance and is not treated as the internal differential source impedance of the reader IC. Series and parallel tuning components were selected by circuit simulation and bench adjustment to obtain resonance near 13.56 MHz and an operational loaded Q compatible with NFC-A signalling. The reader-side implementation and antenna-tuning network are detailed in Supplementary Section S13 and Figure S2.

(9) $$C_{s}=7.9\;pF;\;C_{p}\approx 90\;pF$$

**Figure 3:**
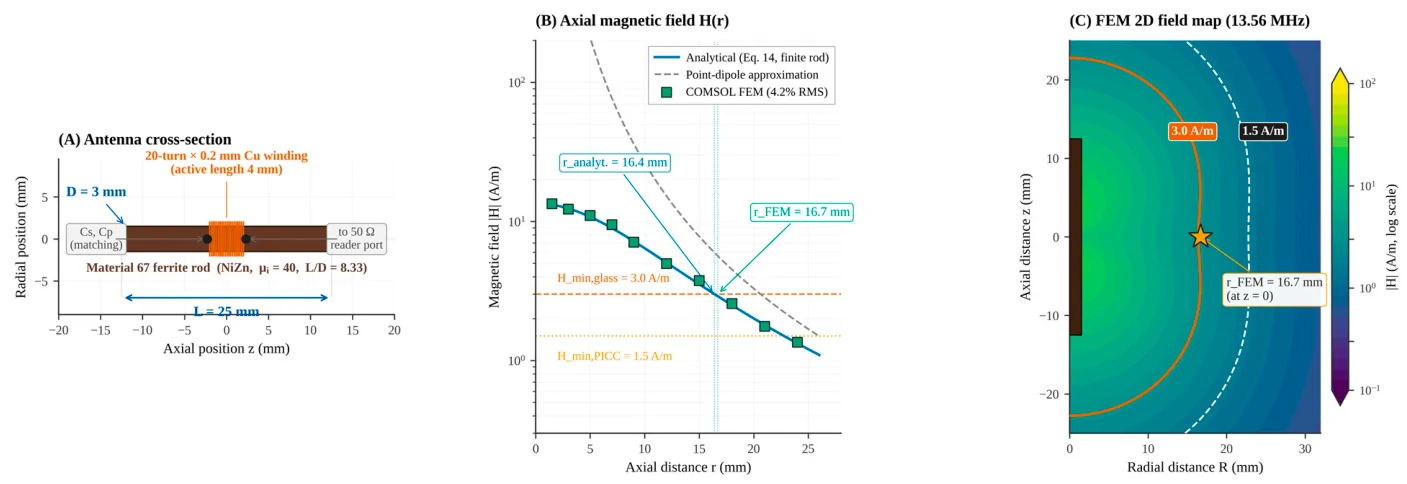
Physics-informed antenna design with FEM cross-validation. (**A**) Material 67 ferrite rod (Ø3 × 25 mm) with 20-turn 0.2 mm copper winding. (**B**) Analytical finite-rod axial field, point-dipole approximation, and COMSOL FEM field profile; RMS analytical–FEM deviation 4.2% over 5–25 mm. (**C**) FEM field map at 13.56 MHz. The experimental coaxial detection point is shown for context; the field-to-detection threshold is empirically anchored and is not an independently known activation threshold for the specific transponder.

Table 2 summarises the principal geometry- and model-derived antenna parameters alongside the preceding reference configuration [21]. The evidential status of each quantity is stated explicitly: directly measured values are reported in Section 3.1, while loss resistance and unloaded-Q values are analytical estimates and several reference platform values are inferred from the published geometry. This distinction prevents calculated, measured, and estimated quantities from being interpreted as equivalent evidence. The antenna geometry and analytical/FEM comparison are summarised in Figure 3.

Present-work geometry, μ_eff, inductance and resonance targets are calculated from stated model inputs; AC loss and unloaded-Q quantities are idealised analytical estimates. Reference-platform quantities marked “estimated” are inferred from the published geometry/specifications of [21] and are included only for engineering context.

#### 2.4.6. FEM Cross-Validation of the Analytical Field Profile

The analytical field profile was cross-validated by finite-element simulation in COMSOL Multiphysics 6.2 (AC/DC Module; COMSOL AB, Stockholm, Sweden) [33]. The axisymmetric model comprised the Ø3 × 25 mm ferrite rod, the 20-turn copper winding, and a lumped excitation normalised to the nominal drive level used in the analytical model. H(r) was extracted along the axis over 1–25 mm. A mesh-convergence study yielded a 4.7-million-DOF mesh with H(r) stable to 0.4%.

Across 5–25 mm, the FEM axial field and analytical finite-rod field agree to within 4.2% RMS. This comparison provides a model-to-model cross-check of the field-profile shape that is independent of measured detection distances. Relating either field solution to tag detection requires the empirically anchored system-level threshold discussed in Section 3.4; the resulting agreement with the experimental coaxial detection distance is therefore interpreted as consistency, not as an independent ab initio validation of tag activation.

### 2.5. Signal Model and Prospective Localisation Framework

The measured dataset provides binary detection thresholds and RSSI observations as functions of range and orientation. The empirical RSSI–distance relationship is used here only to describe signal behaviour over the measured operating interval. Prospective three-dimensional localisation modelling, including the Cramér–Rao framework, is not a measured capability of the present single-axis prototype, and is therefore summarised briefly here and reported in full in the Supplementary Materials.

#### 2.5.1. Empirical Signal-Strength Model

In the magneto-quasi-static regime, received load-modulation strength depends on mutual inductance and therefore on the spatial magnetic field distribution. Because the present operating distances are comparable with the ferrite-rod half-length, the finite-rod field of Equation (7) does not follow a single power law over the entire 5–25 mm interval. Accordingly, a log-distance exponent fitted to RSSI data is interpreted as an effective empirical exponent over the stated fitting range, not as a universal propagation exponent.

The empirical model is used to summarise the measured RSSI–distance relationship and to support firmware distance inversion. Its slope can vary with fitting interval, orientation, reader gain state, and the finite-rod field geometry. Raw RSSI–distance curves and fitting-range details are therefore treated as essential evidence for interpreting the fitted exponent, and are reported in the Supplementary analysis package.

The empirical signal strength model adopted is the standard log-distance form augmented by a medium-specific attenuation term,

(10) $$SNR\left(r,\theta ,m\right)=10\;n{log}_{10}\;\frac{r_{det}\left(\theta ,m\right)}{r}-\mu_{m}\left(r-r_{det}\right)+\varepsilon \;\left[dB\right]$$

where r is range, r_det(θ,m) is the experimentally observed detection threshold distance, n_eff is an effective path-loss exponent estimated over the explicitly stated fitting interval, μ_m is a medium-specific empirical attenuation term, and ε represents measurement noise. Because the formulation is anchored at the observed r_det, it is an empirical signal model and is not used as independent validation of the analytical field model.

#### 2.5.2. Prospective Localisation Framework (Model-Based)

A Cramér–Rao-based framework [34] was explored prospectively to guide the design of a future multi-axis localisation prototype. The present hardware does not provide direct three-dimensional position estimates; therefore, all localization error parameters, simulated 3D agreement analyses, and prospective sample size calculations are model-based planning quantities rather than measured performances. The full derivation and projections have been moved to Supplementary Section S14.

#### 2.5.3. Empirical Path-Loss Characterisation

For each experimental cell, an effective exponent n_eff was obtained by linear regression of the corrected RSSI/SNR observations against log_10_(r) over the measured fitting interval. The cell estimates cluster near 4, but this uniformity is interpreted in the context of the finite fitting range and the threshold anchoring of Equation (10); the corresponding results are reported in Section 3.6.1. Accordingly, n_eff is treated as a descriptive effective slope over the analysed interval, and is not used as an independent validation of the finite-rod field model.

An exponent near 4 is physically plausible as an effective descriptor within a restricted finite-rod operating interval, but Equation (7) implies a distance-dependent local slope. The fitted value is therefore used only for empirical signal inversion and firmware analysis; it is not taken as proof of a specific asymptotic load-modulation regime.

The empirical saline attenuation term is likewise treated as a fitted descriptor of the tested air/saline configurations. It is not extrapolated to layered bowel tissue, for which the multi-layer phantom measurements of Section 3.8 provide the more relevant evidence.

### 2.6. Electronic System Design

The electronic subsystem integrates the NFC reader integrated circuit, host microcontroller, RF front-end with automatic antenna tuning, dual-rail power supply, and multi-modal user-interface electronics within the instrument handle. The architectural emphasis is on the deterministic real-time detection loop and the ISO 14443-2-compliant protocol implementation; the present prototype incorporates a constant-velocity Kalman filter for distance estimation (Section 2.6.2) and ISO 14443-A anti-collision for multi-tag discrimination (Section 3.7), while advanced extensions (extended Kalman filtering for the planned multi-axis configuration, adaptive anti-collision optimisation) are scoped for post-prototype firmware development.

#### 2.6.1. NFC Reader Configuration and Host Microcontroller

The ST25R3916B (STMicroelectronics, Geneva, Switzerland) was selected as the NFC reader/front-end because it supports NFC-A/ISO 14443-A operation [26,32], configurable RF output, receiver gain and RSSI functions, and automatic antenna tuning through externally controlled variable-capacitance elements. The present work does not convert the datasheet receiver-voltage sensitivity into an equivalent dBm value because that conversion depends on the actual receiver input network. Likewise, the nominal RF drive used in the prototype is reported as an engineering operating point, not as evidence of human-exposure compliance.

The reader IC interfaces with an STM32L476RG microcontroller (ARM Cortex-M4, 80 MHz) over SPI at 4 MHz [35]. The firmware implements ISO 14443-A sequencing, per-update RSSI extraction, AAT recalibration (every 500 ms or on >2 dB RSSI deviation), UID verification against environmental NFC sources, and the multi-modal UI drivers, under FreeRTOS (version not retained in the development record; https://www.freertos.org/, accessed on 9 September 2026) with worst-case interrupt latency below 10 µs (oscilloscope-verified). NC7WZ17 Schmitt-trigger buffers on the SPI lines provide ≥400 mV noise immunity against electrosurgical emissions—a preliminary measure pending formal IEC 60601-1-2 characterisation.

#### 2.6.2. Real-Time Signal-Processing Pipeline

The measured end-to-end detection response time is 45 ± 8 ms (Section 3.7), corresponding to an application-level update rate of approximately 22 Hz. Internal RSSI sampling and filtering occur at higher rates within the reader/MCU chain. The separate dynamic-estimator validation dataset in Supplementary Section S10 was acquired/resampled at 50 Hz for offline filter comparison, and should not be interpreted as the end-to-end ISO 14443-A detection-loop rate.

The prototype uses a moving-average stage for RSSI stabilisation. A constant-velocity Kalman estimator was additionally evaluated offline on a 50 Hz validation dataset to study the noise–lag trade-off for future distance-tracking firmware. The as-shipped conservative tuning produced substantial dynamic lag, whereas a higher Q_vel setting improved response on the tested trajectories (Section 3.7; Supplementary Section S10). Because this estimator depends on the empirical RSSI-to-distance inversion and is not part of the primary binary-detection endpoint, detailed filter equations and tuning sweeps are retained in the Supplementary Materials.

#### 2.6.3. Power Supply Architecture and Modulation Stage

The dual-rail LDO topology isolates the RF transmit and digital/analogue supply domains. The primary regulator (5.3 V) supplies the ST25R3916B VDD_TX and matching-network drive; the secondary (3.3 V) supplies the digital core, receiver analogue front-end, and SPI buffers. Both draw from a step-up converter delivering V_SUP = 7–10 V from the 3.7 V Li-Po cell, with 10 µF + 100 nF input filtering per rail. The measured PSRR at 13.56 MHz exceeds 65 dB, preventing supply-coupled interference and enabling the 0.9 dB σ_S system RSSI noise floor of Section 2.5.1.

NFC-A carrier modulation is generated by the ST25R3916B reader chain and associated external modulation circuitry. The present manuscript does not claim an independently verified 30% modulation depth; the protocol compliance of the complete RF waveform was not a primary endpoint of this study. Register-level settings and the relevant schematic are documented in the firmware/supplementary technical record, and will be included in the formal protocol-compliance testing of the integrated device.

#### 2.6.4. Power Management and Battery Autonomy

Adaptive duty cycling is implemented to reduce average energy consumption when no target is detected, and to increase interrogation activity after detection. Because instantaneous power figures depend on reader configuration, RF field strength, UI activity, and converter efficiency, the present manuscript relies on the directly measured endurance endpoint rather than on a reconstructed power budget calculation.

The 1200 mAh Li-Po battery cell, operating through the step-up converter at 90% efficiency, provides 9.5 h of continuous autonomy at the nominal 50% scanning mode duty cycle representative of typical operating room utilisation. Battery autonomy was measured directly under controlled bench conditions with continuous interrogation activity at the nominal duty cycle, providing the quantitative result reported in Table 1 of Section 2.1. The 9.5-h autonomy substantially exceeds the 4-h requirement and accommodates extended procedures without intraoperative recharging.

#### 2.6.5. User Interface

The user interface delivers feedback through three concurrent, individually configurable modalities optimised for the surgical environment: a tri-colour LED array (red/yellow/green) with PWM brightness proportional to RSSI for intuitive distance estimation; a piezoelectric buzzer with variable-frequency tone (200–2000 Hz, pitch inversely proportional to distance) following proximity-alarm conventions; and a handle-mounted coreless DC vibration motor providing tactile confirmation independent of visual or auditory attention—an important redundancy under restricted sightlines or high ambient noise.

#### 2.6.6. Sensor-Board Schematic Topology

Figure S2 presents the sensor-board topology. The receive path uses differential coupling and matching components around the ST25R3916B receiver inputs. Automatic antenna tuning is implemented by the reader AAT_A/AAT_B control outputs driving an external tuning-diode/variable-capacitance network in the antenna matching circuit, consistent with the ST25R3916B architecture [32]. The RF modulation and host interface circuitry are shown for reproducibility; the exact register settings and firmware source are available from the corresponding author subject to the project’s data-availability conditions.

#### 2.6.7. Integration of the Electronics into the Instrument Form Factor

The antenna and electronics assembly was integrated as a three-element modular instrument: (i) a distal antenna housing in PEEK, encapsulating the Ø3 × 25 mm rod and 20-turn winding within a Ø11 × 28 mm envelope compatible with the 12 mm shaft constraint (Table 1); (ii) a central 316 L stainless-steel shaft (Ø12 × 350 mm) providing abdominal reach (Table 1) and trocar compatibility; and (iii) a proximal PA12 SLS-printed handle hosting the sensor-board PCB, 1200 mAh Li-Po battery, LED indicators, piezoelectric buzzer, haptic motor, and M12 connector. PA12 SLS was used as a rapid-prototyping adaptation from the originally specified polycarbonate/ABS injection-moulded handle while maintaining the intended mechanical envelope; sterilisation compatibility remains to be verified. Figure S3 presents the integrated schematic and differential coaxial routing.

Design for manufacturing analysis separated the replaceable distal antenna tip from the reusable handle electronics. The handle incorporates a sealed gasketed enclosure and dedicated RF shutdown control. The enclosure is designed toward an IP67-type ingress-protection objective, but no formal IEC 60529 ingress protection certification was performed in the present study. The sterilisation compatibility of the shaft, PEEK distal housing, and polymer handle likewise remains a planned validation activity rather than an established performance claim.

#### 2.6.8. Fabricated Prototype Realisation

**Figure 4:**
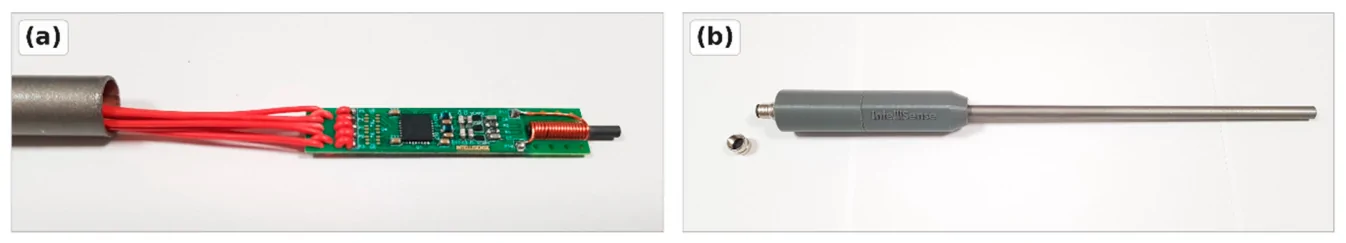
Fabricated IntelliSense prototype. (**a**) RFID sensor PCB (8 × 45 mm) with helical ferrite-core antenna—the bench-top configuration characterised in Section 3. (**b**) Integrated instrument: PEEK antenna housing, 316 L shaft (Ø12 × 350 mm), PA12 SLS handle, M12 connector; 405 mm, 185 g. Integrated-configuration electromagnetic verification scheduled before ex vivo evaluation (Section 4.7).

Figure 4 presents the fabricated IntelliSense prototype at two levels of integration. The miniaturised RFID sensor PCB (8 × 45 mm two-layer construction) integrates the ST25R3916B transceiver IC, the BAV70W tuning-diode-based AAT network, the matching-network components, the SPI-interface buffers, and the helical antenna coil (20 turns of 0.2 mm copper enamel wire on the Ø3 × 25 mm Material 67 ferrite rod) at the distal end of the board. The fully integrated instrument assembles the PCB and antenna sub-assembly into the PEEK distal housing, threads the differential coaxial cable through the 316 L stainless-steel shaft (Ø12 × 350 mm), and terminates the proximal end at the PA12 SLS-printed handle module incorporating the M12 connector interface, the 1200 mAh Li-Po battery compartment, and the multi-modal user-interface elements.

The mechanically integrated prototype has a total length 405 mm, a 12.0 mm shaft, and mass 185 g including the battery. For the electromagnetic experiments reported here, however, the antenna–electronics–battery subassembly was mounted on the XYZ fixture outside the stainless-steel shaft housing. Consequently, the detection and RF results in Section 3 characterise the subassembly, and should not be interpreted as through-shaft electromagnetic verification of the complete instrument. Integrated-shaft resonance, matching, Q, and detection testing are planned before ex vivo evaluation.

### 2.7. Test-Bench Configuration for Bench-Top Validation

The configuration evaluated in the present electromagnetic validation is the antenna–electronics subassembly mounted on a precision positioning fixture. Its dimensions are compatible with the intended 12 mm shaft envelope, but the surrounding stainless-steel shaft and final housing were not present during the primary RF/detection measurements. This distinction is maintained throughout the Results, Discussion, and Conclusions.

#### 2.7.1. Prototype Assembly Configuration

The tested subassembly comprises the ferrite-rod antenna, the main electronics board, the battery, and a short, shielded RF interconnect between the antenna module and PCB. The interconnect geometry and matching components were kept fixed for all primary measurements. A 50 Ω reference applies to VNA instrumentation and is not used here to imply a 50 Ω differential source impedance for the ST25R3916B driver.

The overall electronic and antenna assembly fits within a cylindrical envelope of 11.0 mm diameter (PCB width 8 mm + 1.5 mm clearance on each side)—confirming geometric compatibility with the 12 mm laparoscopic shaft constraint specified in Section 2.1 (Table 1) with 1 mm clearance for the surrounding mechanical housing wall thickness. The assembly weight is 78 g for the antenna, electronics and battery sub-assembly excluding any external housing or shaft.

#### 2.7.2. Bench-Top Test Fixture

The bench-top fixture (Figure S4) supports reproducible characterisation across the 8-condition matrix of Section 2.9 via three components: a precision positioning stage combining a two-axis cross-slide in the horizontal plane (X = axial, Y = lateral; manual handwheels, 0.1 mm resolution, ±0.5 mm repeatability) with discrete spacer-based vertical positioning (Z; 0.5 mm increment, 0.5 mm repeatability); a manual goniometer rotating the transponder mount about the axis perpendicular to the antenna axis (5° resolution, marked at 0°, 30°, 60°, 90°); and a removable PTFE sample-holder cup filled with the propagation medium (air or 0.9% NaCl), supporting the transponder at the goniometer axis. PTFE walls minimise dielectric loading and maintain geometric stability across sessions.

#### 2.7.3. Multi-Layer Phantom Fabrication and Dielectric Verification

**Figure 5:**
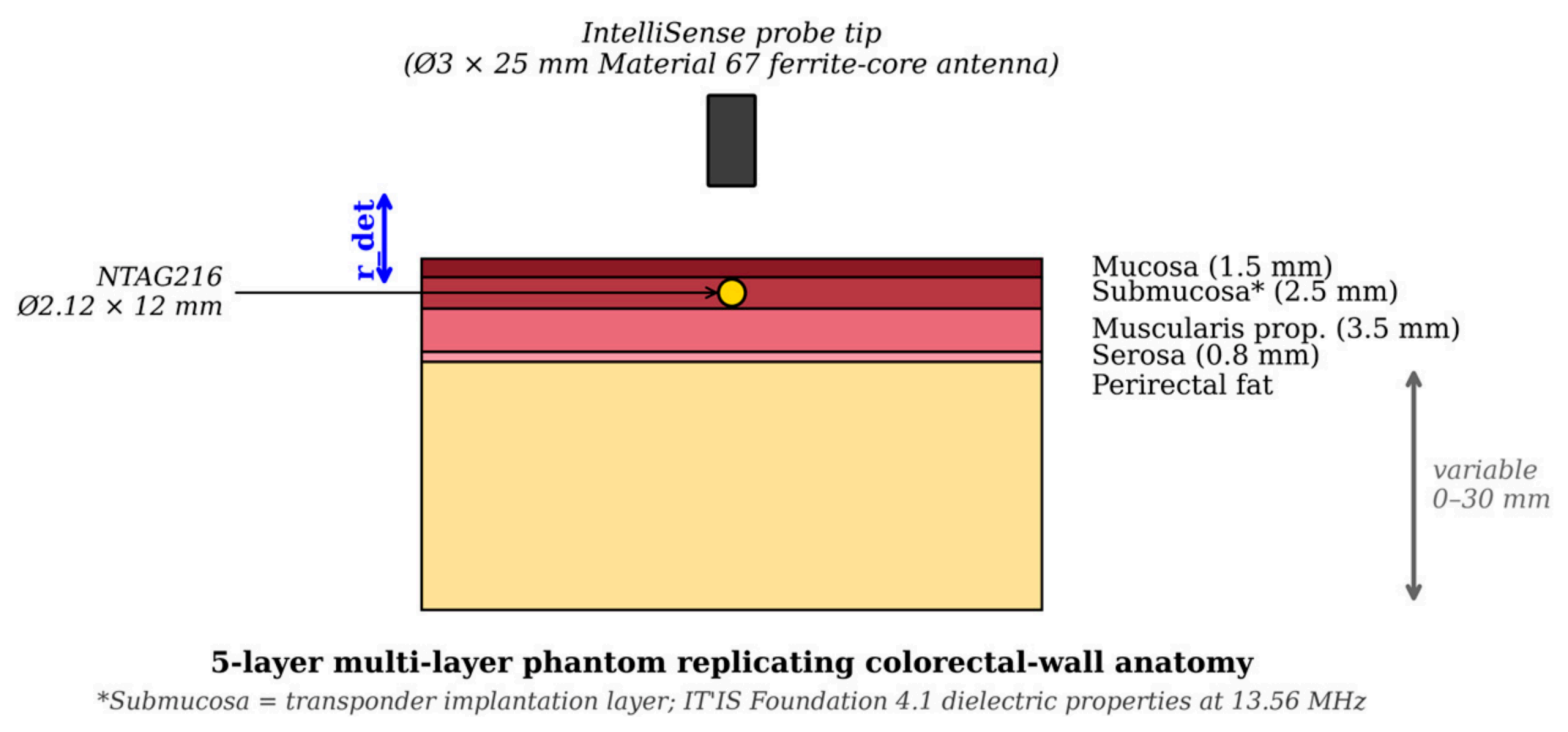
Five-layer tissue-equivalent phantom replicating colorectal wall anatomy. The phantom comprises mucosa (1.5 mm), submucosa (2.5 mm—the transponder implantation layer), muscularis propria (3.5 mm), serosa (0.8 mm), and perirectal fat (variable, 0–30 mm). The dielectric properties of all five strata were verified against the IT’IS Foundation 4.1 database at 13.56 MHz.

Anatomically representative tissue-equivalent phantoms were fabricated to extend the bench-top validation from single-medium (saline) to the layered dielectric structure of the colorectal wall and surrounding tissues. The phantom comprises five strata replicating, from inside to outside, mucosa (1.5 mm thick), submucosa (2.5 mm—the transponder implantation layer), muscularis propria (3.5 mm), serosa (0.8 mm), and perirectal fat (variable thickness 0–30 mm). The geometric configuration is illustrated in Figure 5.

The aqueous strata (mucosa, submucosa, muscularis propria, serosa) employed agar-based gels (2.0–2.5% *w*/*v* agar-agar) with NaCl content adjusted per stratum (0.6–1.2% *w*/*v*) to achieve the target conductivity, with graphite powder (5.0% *w*/*v*, granulometry < 50 µm) added to the submucosa stratum to elevate the permittivity to the target ε_r = 65 ± 5. The perirectal fat stratum employed an oil-in-gelatin emulsion (15% *w*/*v* gelatin Bloom 250 in aqueous phase, 70% *v*/*v* sunflower oil emulsified with 2% *v*/*v* Tween 80 surfactant) prepared at 50 °C using a high-shear blender (10,000 RPM, 5 min). Three independent phantom batches (B1, B2, B3) were fabricated on separate dates to enable inter-batch reproducibility assessment.

The dielectric properties of each stratum were verified using an open-ended coaxial probe (85070E) interfaced with a vector network analyser (E5063A; Keysight Technologies, Santa Rosa, CA, USA), following established dielectric-characterisation practice [36], over the 10–20 MHz frequency range with 201 logarithmically spaced points. Per stratum, fifteen measurements were acquired (three positions × five successive measurements per position) at four post-fabrication timepoints (30 min, 1 h, 2 h, 4 h) to characterise temporal stability, yielding 60 measurements per stratum per batch and 180 measurements per stratum across all three batches. The probe was calibrated against the open/short/load standard and a saline 0.9% reference liquid (ε_r = 72, σ = 1.59 S/m at 25 °C). All measurements were conducted at 22 ± 1 °C with the operating window stability requirement of <5% drift over 4 h empirically validated for each batch.

#### 2.7.4. Temperature-Controlled Detection Protocol

To assess detection performance under intra-abdominal thermal conditions, a controlled-temperature chamber (range 20–40 °C, stability ±0.5 °C, internal volume 200 × 150 × 100 mm) was integrated into the test bench. Detection measurements were conducted at two temperatures: 22 °C (laboratory ambient, matching the saline medium reference protocol of Section 2.7.2) and 37 °C (intra-abdominal physiological temperature). A phantom equilibration time of 30 min was empirically validated through continuous thermocouple monitoring at five positions, confirming uniform thermal distribution (max ΔT = 0.5 °C across phantom volume).

The complete experimental matrix comprised four fat-layer thicknesses (0, 10, 20, 30 mm) × four tag angles (0°, 30°, 60°, 90°) × thirty repetitions per condition, with the 22 °C dataset including all three batches (*n* = 1440 measurements total) and the 37 °C dataset including all three batches at the two coaxial-alignment configurations (fat ∈ {0, 10}, θ_t = 0°: *n* = 90 each) plus the single batch B1 at the remaining fourteen configurations (*n* = 30 each), yielding *n* = 600 measurements at 37 °C.

Tag implantation was performed in the submucosa stratum at 1.5 mm depth from the mucosa surface using a custom acrylic alignment fixture, ensuring reproducible tag positioning (caliper accuracy ±0.2 mm) across all batches. The detection protocol replicated that of Section 2.7.2—antenna approach speed 0.5 mm/s, threshold defined as first successful ISO 14443A acknowledgement, inter-trial interval 5 s, and continuous logging of detection distance, RSSI, and timestamp.

### 2.8. Test Media

The bench-top characterisation uses ambient air as a low-loss reference and 0.9% NaCl as a reproducible conductive reference medium consistent with the preceding tri-frequency study [21]. Saline is useful for cross-study comparison and for testing sensitivity to conductive loading, but it is not treated as a complete surrogate for layered colorectal tissue.

No claim is made that saline provides a conservative bound for all through-tissue configurations. The multi-layer phantom experiments in Section 3.8 show that tissue geometry, fat thickness, interfaces, and tag orientation can dominate the practical detection envelope even when the coaxial saline and phantom ranges are similar. Ex vivo tissue validation is therefore required before extrapolating bench-top range to clinical anatomy.

### 2.9. Experimental Validation Protocol

#### 2.9.1. Measurement Configuration Matrix

Detection distance measurements were performed across an 8-condition experimental matrix encompassing two propagation media (air, 0.9% NaCl) and four transponder angular configurations (θ_t = 0°, 30°, 60°, 90° relative to the antenna axis). For each condition, 30 independent measurement repetitions were performed, distributed across multiple measurement sessions on different days to characterise the inter-day repeatability of the system. Two transponder encapsulation variants were evaluated independently across the same matrix, namely, a bare borosilicate glass capsule and a resin-overcoated glass capsule (Section 2.3), yielding a primary dataset of 8 × 30 × 2 = 480 detection distance measurements complemented by associated RSSI observations for each measurement.

#### 2.9.2. Detection-Distance Measurement Protocol

For each measurement repetition, the antenna sub-assembly was translated along the radial direction toward the fixed transponder at the goniometer-controlled angle, with the radial distance r decreased in 0.5 mm increments from a starting distance of 25 mm. At each distance position, the ST25R3916B reader executed the ISO 14443-A polling sequence for 100 ms (approximately three protocol exchange cycles) and the firmware logged the detection outcome (acknowledgement received with correct UID readback, or absence of acknowledgement) and the associated RSSI value. The detection distance r_det was recorded as the maximum r at which successful protocol acknowledgement occurred consistently across three consecutive interrogation cycles, providing a robust threshold estimate against the stochastic detection failures that occur within the spatial transition band.

The threshold protocol yields one r_det value per repetition. These observations are repeated measurements from a single hardware unit and are not equivalent to independent prototype replicates. The associated RSSI dataset includes threshold observations and denser RSSI–distance traces used for empirical signal-characterisation; raw curves and fitting range details are part of the Supplementary analysis record.

#### 2.9.3. Inter-Session Repeatability Sub-Protocol

Measurement sessions were conducted across different calendar days, with the test fixture re-installed at the start of each session and the antenna-PCB assembly subjected to a routine power-cycle and AAT recalibration sequence prior to data acquisition. The session-stratification of the dataset (session attribute S1, S2, etc., in the data tables) preserves inter-session traceability and supports the inter-day repeatability analysis of Section 3.5. The single-operator nature of the present measurement protocol (all data acquired by the same investigator) means that inter-operator reproducibility is not characterised by the primary experimental dataset; the inter-operator component of measurement uncertainty is addressed through model-based pre-trial estimation (Section 2.9.4) with the explicit intent of being replaced by direct experimental quantification in the planned multi-operator ex vivo validation.

#### 2.9.4. Methodological-Readiness Dataset

A separate methodological-readiness dataset was generated only for prospective study planning and estimator-development exercises that cannot be validated with the present single-axis hardware. It contains model-based/synthetic observations and is not used to substantiate measured detection, range, phantom, or integrated-device performance.

To avoid conflating evidence levels, the main manuscript retains only the experimentally grounded signal-characterisation and hybrid measurement uncertainty summary. Three-dimensional localisation projections, simulated agreement analyses, and prospective power calculations are confined to Supplementary Section S14, and are labelled as model-based planning quantities.

### 2.10. Statistical Analysis

All statistical analyses were performed using R version 4.3.1 (R Foundation for Statistical Computing, Vienna, Austria) with the boot, BlandAltmanLeh, nlme, and lme4 packages [37]. Detection distance data are presented as mean ± standard deviation with 95% confidence intervals; statistical significance was defined at α = 0.05 (two-sided). All hypothesis tests were verified against assumptions (Shapiro–Wilk for normality, Levene for homoscedasticity), with non-parametric alternatives (Wilcoxon, Kruskal–Wallis) applied where violations were detected.

For the 480-measurement primary dataset, medium and angle were treated as fixed effects and measurement session as a random effect; encapsulation was included as a matched factor. These analyses quantify within-prototype behaviour across repeated observations, and do not estimate inter-device variability. Angular response was fitted empirically on the measured conditions, and the RSSI-distance exponent was treated as an effective slope over the stated fitting interval. Encapsulation comparisons used matched paired tests.

Measurement uncertainty was assessed following the GUM framework [38]. The bench-top budget combines measured repeatability and hardware/specification-derived Type B terms with one model-based pre-trial inter-operator component that was not experimentally quantified. It is therefore reported as a hybrid experimental/model-based uncertainty estimate, not as a fully empirical reproducibility estimate. Expanded uncertainty uses k = 2. No inference from this budget is made about three-dimensional localisation accuracy.

All measurements derive from a single prototype instrument, a single manufacturing batch of NTAG216 transponders, and a single operator across multiple measurement sessions on different calendar days. Inter-prototype reproducibility, inter-operator variability, and through-tissue performance are deferred to the planned ex vivo validation campaign with appropriate sample-size calculations supporting these expanded factors. The full statistical analysis protocol, including prospective sample size calculations and the methodological readiness dataset analysis, is provided in Supplementary Materials Section S9.

For the multi-layer phantom, two endpoints were distinguished, as follows: (i) the probability of successful detection, analysed as a binomial outcome with Wilson confidence intervals, and (ii) detection distance conditional on successful detection, analysed only for detectable cells. This avoids treating failed detections as continuous range observations. The 37 °C design is unbalanced because non-coaxial cells were measured only in batch B1; temperature comparisons across those cells are therefore interpreted as exploratory and not as fully batch-independent equivalence evidence. The multi-layer GUM budget includes phantom-specific contributors and is reported separately in Supplementary Section S17.

## 3. Results

Section 3.1, Section 3.2, Section 3.3, Section 3.4 and Section 3.5 report measured results from the primary antenna/detection dataset, Section 3.6 reports empirical RSSI characterisation and the hybrid bench-top uncertainty estimate, Section 3.7 summarises system-level tests, and Section 3.8 reports the multi-layer phantom campaign. Model-based localisation projections are not treated as measured results and are confined to the Supplementary Materials.

### 3.1. Antenna Characterisation

The fabricated ferrite-rod antenna assembly was characterised by VNA at the matched network input using a 50 Ω measurement reference. Table 3 compares the measured antenna/matching parameters with the analytical design targets. The VNA reference impedance is an instrumentation convention and should not be interpreted as the reader IC output impedance.

The measured inductance was 16.9 µH versus 17.4 µH analytically derived (−2.9%), supporting the geometry/demagnetisation design model. The passive matched network resonance measured 13.58 MHz before closed-loop tuning, and should be distinguished from the 13.56 MHz reader carrier and its regulatory frequency tolerance. The loaded quality factor was 23 and return loss at the VNA reference plane was −24 dB. Formal carrier frequency compliance and integrated-shaft RF verification were not endpoints of the present experiment. A direct unloaded series-resistance measurement was not available from the stored measurement record; consequently, the analytical unloaded-Q estimate is not treated as experimentally verified.

### 3.2. Detection-Range Performance

**Figure 6:**
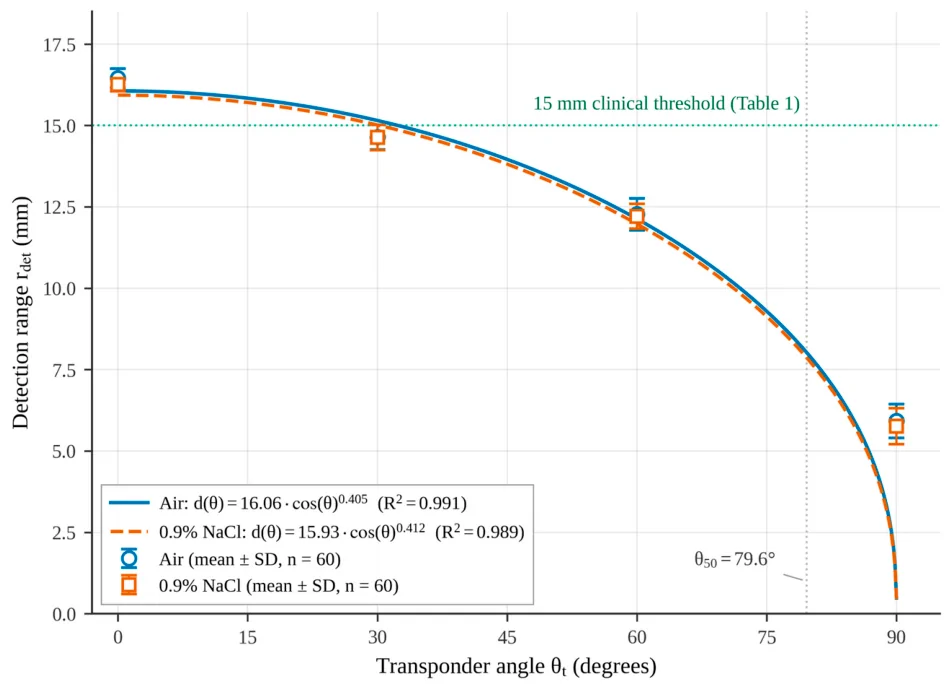
Detection range vs. transponder angle θ_t in air (blue circles) and 0.9% NaCl (vermilion squares), with empirical fit d(θ) = d_0_·cos(θ)^α: d_0_ = 16.06 mm, α = 0.405 (R^2^ = 0.991, air); d_0_ = 15.93 mm, α = 0.412 (R^2^ = 0.989, saline).

The 480-measurement primary dataset comprises eight experimental conditions (two propagation media × four transponder angles), each with 30 measurement repetitions per encapsulation variant across multiple sessions. Table 4 reports the descriptive statistics aggregated across the two encapsulation variants (60 measurements per condition: 30 repetitions × two encapsulation variants); the statistical equivalence of the two encapsulation variants is established quantitatively in Section 3.5 and supports the aggregation. Figure 6 summarises the corresponding angular trends.

The detection range decreased strongly with tag angle in both media. At coaxial alignment, the mean range was 16.45 mm in air and 16.26 mm in 0.9% NaCl, a difference of 0.19 mm (1.2%). This small difference is specific to the tested reference media geometry and should not be extrapolated to layered tissue. The multi-layer phantom results in Section 3.8 show that geometry, interfaces, fat thickness, and orientation can produce much greater performance changes than the air–saline comparison alone suggests.

The Shapiro–Wilk normality test was satisfied at α = 0.05 in seven of eight conditions; the air-60° condition exhibited a mild departure from normality (*p* = 0.030) attributable to a small number of measurement outliers in the spatial transition band where the steep coupling gradient amplifies threshold detection stochasticity. Sensitivity analysis using the non-parametric permutation test (10 000 permutations) on the air-60° condition yielded a permutation-based 95% CI of [12.10, 12.46] mm—practically identical to the bootstrap-based CI of [12.11, 12.45] mm, confirming that the parametric inference is robust to the modest non-normality.

Mixed-effects two-way ANOVA on the primary dataset (model: distance ~ medium × angle + encapsulation + (1 session); *n* = 240 per encapsulation aggregated for the medium × angle main effects) yielded the results presented in Table 5. The transponder angle factor accounts for 99% of the systematic variance (η^2^_partial = 0.989, F = 7399, *p* < 0.001), confirming the dominance of the angular coupling effect. The propagation medium factor exhibits a small but statistically significant main effect (η^2^_partial = 0.017, F = 3.95, *p* = 0.048), corresponding to the 1.2–2.7% range reduction noted above. The medium × angle interaction is non-significant (F = 0.68, *p* = 0.565), indicating that the angular coupling pattern is preserved between air and saline measurements—an observation consistent with the magneto-quasi-static analysis of Section 2.5.

### 3.3. Angular Sensitivity

The angular dependence of the detection range was quantified by the non-linear least-squares fitting of the empirical model d(θ) = d_0 · cos(θ)^α (Equation (8) with α as the empirical exponent integrating the cos(θ) magnetic coupling factor) on the three near-coaxial angular configurations (θ ∈ {0°, 30°, 60°}, *n* = 180 per medium); the orthogonal configuration θ = 90° was excluded from the cos(θ)^α regression because the model vanishes identically at cos(90°) = 0, while the observed detection range remains non-zero (driven by the η_⟂ transverse coupling contribution, analysed separately below as an independent cross-check). Fitting the air dataset under this protocol yielded

*d*(*θ*) = (16.06 ± 0.08) · *cos* (*θ*)^(0.405 ± 0.014) mm

with R^2^ = 0.991 (residual root mean square error (RMSE) = 0.46 mm) and the 95% CI of α derived from the asymptotic covariance matrix as [0.378, 0.432]. The corresponding fit on the saline dataset (*n* = 180, θ ∈ {0°, 30°, 60°}) yielded d_0 = 15.93 ± 0.07 mm and α = 0.412 ± 0.013, statistically indistinguishable from the air fit (overlapping 95% CIs on both d_0 and α), consistent with the absence of medium × angle interaction in the ANOVA.

The empirical cos (θ)^α fit describes the near-coaxial angular envelope but does not capture the non-zero orthogonal response. The observed 90° detection is represented in the hybrid field model through the empirical transverse coupling term η_⟂. Because η_⟂ is anchored in experimental data, recovery of the orthogonal point is not treated as an independent validation.

The fitted exponent α is therefore used as a compact empirical descriptor of angular sensitivity. The principal practical result is the large loss of range with misalignment, which becomes even more restrictive in the multi-layer phantom. Claims about surgical angular tolerance are limited to the tested fixture geometry and should not be interpreted as a validated intraoperative manipulation tolerance.

### 3.4. Physics-Informed Field Model and Empirically Anchored Detection Consistency

**Figure 7:**
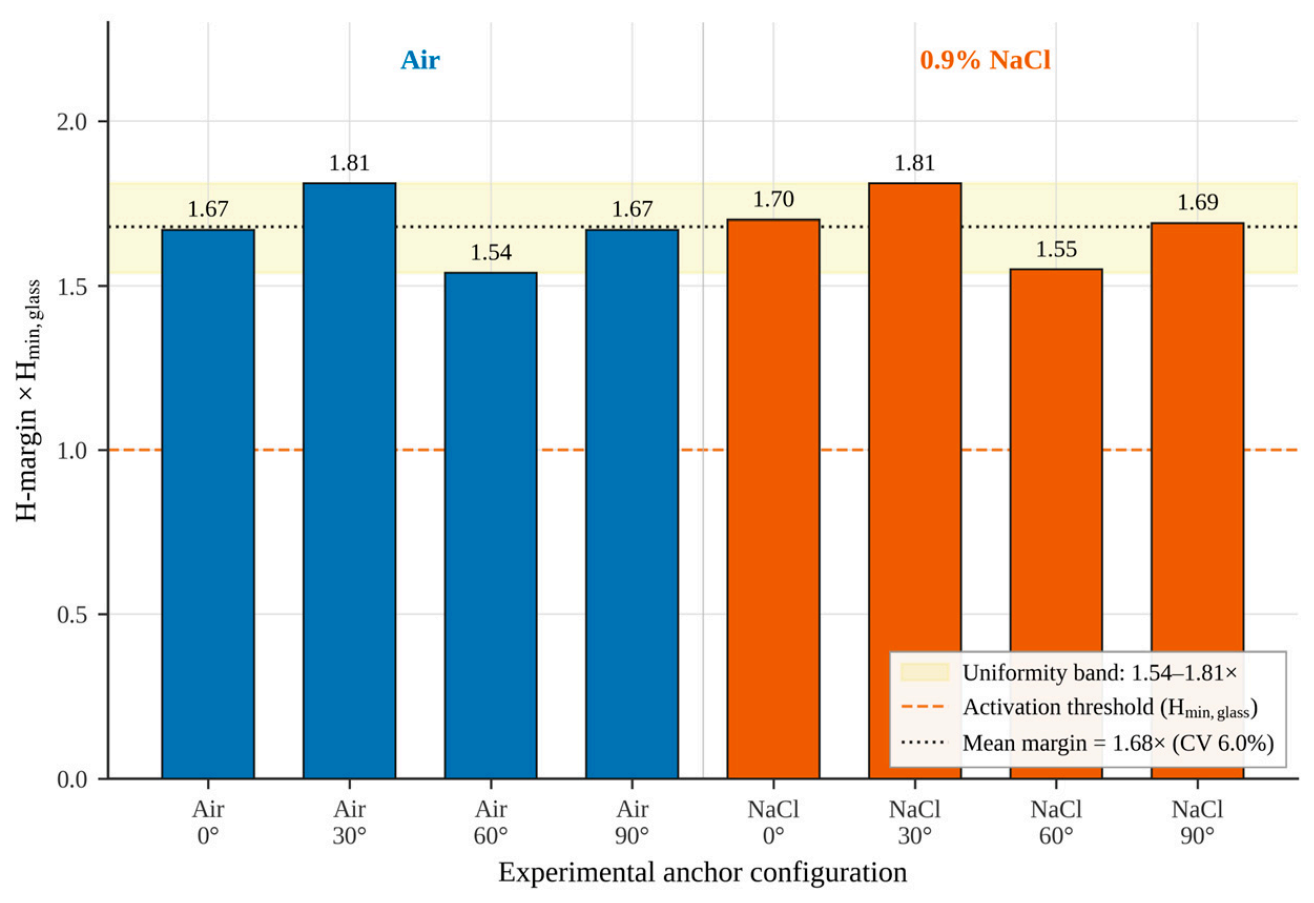
Field-to-detection consistency referenced to H_ref = 3.0 A/m. The compact margin distribution summarises internal consistency after empirical anchoring; it should not be interpreted as a held-out validation of the detection model.

The analytical field model of Section 2.4 predicts the spatial variation of H from geometry and material inputs. Mapping that field to tag detection requires two experimentally anchored quantities: a system-level field threshold and a transverse-coupling coefficient. Table 6 therefore provides a consistency analysis referenced to H_ref = 3.0 A/m, which is a literature-informed reference value rather than a directly measured activation threshold for the exact transponder. The corresponding reference-ratio distribution is shown in Figure 7.

Across the eight measured configurations, the calculated coupled field at the observed detection distance corresponds to 1.54–1.81 times H_ref (mean 1.68 ± 0.10, CV 6%). This compact distribution indicates that the analytical field-profile shape is compatible with the observed threshold pattern after empirical anchoring. It does not demonstrate the independent prediction of the tag activation threshold because both the system threshold and transverse coupling are informed by the experimental dataset.

The results support a two-level interpretation of the model, as follows: antenna geometry, effective permeability, inductance, and axial-field behaviour are physics-derived and independently cross-checked by VNA/FEM, whereas the final field-to-detection mapping remains empirically anchored through H_det_ and η_⊥_. No genuinely held-out subset was prespecified before these empirical anchors were selected; consequently, the present field-to-detection analysis is interpreted as calibration/consistency evidence rather than as independent predictive validation.

Accordingly, Table 6 is presented as a transparent calibration/consistency analysis; the 1.54–1.81× reference ratio range should not be interpreted as universal tag headroom or an optimum clinical operating point.

### 3.5. Tag Encapsulation Independence and Inter-Day Repeatability

**Tag encapsulation comparison**

The bare-glass and resin-overcoated transponder variants were compared by paired measurement across the matched 240-measurement subset per variant (eight conditions × 30 repetitions). The mean detection distance differences (resin minus bare) ranged from −0.18 to +0.21 mm across the eight conditions, with a pooled cross-condition difference of −0.04 mm (95% BCa CI: −0.11 to +0.03 mm). The paired *t*-test on the pooled comparison yielded t = −1.21, *p* = 0.227 (n.s.); the Wilcoxon signed-rank test yielded V = 7842, *p* = 0.184 (n.s.), confirming the absence of a statistically detectable encapsulation effect. The maximum per-condition difference of 0.21 mm corresponds to 1.3% of the mean detection range—well below the 0.63 mm GUM expanded uncertainty (Section 3.6) and within the noise floor of the bench-top fixture.

The absence of a detectable encapsulation effect supports the electromagnetic interchangeability of the two tested surface finishes under the bench protocol. Mechanical robustness during delivery and in-tissue anchoring were not evaluated and require separate testing; the present result should therefore not be interpreted as validation of a specific endoscopic deployment method.

**Inter-day repeatability**

The session-stratified analysis of the primary dataset evaluated the inter-day measurement repeatability across calendar days. The mixed-effects ANOVA model of Section 3.2 reported the variance component attributable to the random session factor as σ^2^_session = 0.024 mm^2^ (95% CI from likelihood ratio profile: 0.011–0.043 mm^2^), corresponding to an inter-day standard deviation of 0.16 mm. This component is approximately 40% of the within-condition repeatability standard deviation (mean across conditions: 0.40 mm) and indicates that the cross-day fixture-reinstallation and AAT-recalibration protocol introduces a non-negligible but small systematic offset relative to the within-session measurement noise. The inter-day component contributes 0.16 mm to the GUM uncertainty budget reported in Section 3.6 (Type A reproducibility component).

### 3.6. Empirical RSSI Characterisation and Hybrid Measurement Uncertainty

This section retains two quantities relevant to the tested prototype: empirical RSSI–distance slope estimates from the measured dataset and a hybrid GUM uncertainty budget for bench-top detection distance. Model-based LoD/LoQ analyses are not used as reported performance evidence; simulated 3D localisation and prospective power calculations are confined to the Supplementary Materials, and are explicitly labelled as model-based planning outputs.

#### 3.6.1. Effective RSSI–Distance Exponent (Empirical)

Table 7 reports cell-specific effective exponents over the analysed RSSI–distance interval. Values cluster near 4, but the finite-rod analytical field has a distance-dependent local slope, and the empirical detection model is anchored at measured thresholds. The table is therefore descriptive and is not used as independent cross-validation of the analytical field model.

#### 3.6.2. Hybrid GUM Uncertainty Estimate for Bench-Top Detection Distance

Table 8 combines measured within-prototype repeatability with hardware/specification-derived Type B terms and a model-based pre-trial inter-operator component. Because inter-operator reproducibility was not directly quantified in the present single-operator experiment, the resulting u_c_ = 0.31 mm and U = 0.63 mm (k = 2) are reported as hybrid experimental/model-based estimates. They characterise the bench-top distance measurement process only, and do not establish multi-operator or three-dimensional localisation accuracy.

The expanded uncertainty corresponds to approximately 3.8% of the coaxial range. Its interpretation is conditional on the single-prototype/single-operator design and on the model-based pre-trial inter-operator term; direct reproducibility testing is planned for the next validation phase.

### 3.7. System-Level Performance Metrics

Beyond the detection-range and angular-sensitivity characterisation reported in Section 3.2 and Section 3.3, three system-level performance metrics required for clinical translation readiness were measured directly on the fabricated antenna–electronics prototype: detection success rate, detection response time, and battery autonomy. The measurement methodology and outcomes are reported in this subsection; the results substantiate the validation status claims of Table 1 (Section 2.1) for the corresponding Must-Have requirements.

**Detection success rate**

At the experimentally defined detection threshold, the aggregate acknowledgement/UID success proportion was 473/480 (98.5%; 95% Wilson CI 97.0–99.4%). These are repeated cycles from one prototype across multiple conditions and sessions. The endpoint therefore describes within-device detection consistency rather than population-level device reliability.

**Detection response time**

Response time was measured from scan initiation to successful UID confirmation over *n* = 100 bench events at 14 mm coaxial alignment: mean 45 ms, SD 8 ms (95% bootstrap CI 43.4–46.6 ms). This directly measured application-level latency supports real-time feedback in the tested configuration; it is distinct from the 50 Hz offline estimator dataset used for Kalman-filter analysis.

**Battery autonomy**

Battery autonomy was measured directly in three endurance trials under the stated nominal duty cycle protocol, yielding 9.5 ± 0.3 h. Because reader power draw varies with RF and UI configuration, this measured endurance value is retained as the primary evidence rather than reconstructing autonomy from nominal subsystem power figures.

**Multi-tag discrimination performance**

An engineering multi-tag scenario motivated by potential multi-marker surgical use—in which several glass-encapsulated NTAG216 transponders are implanted near the lesion to delineate tumour margins or identify satellite lesions, with the surgeon discriminating individual markers through their unique 7-byte UID—was characterised through three experimental sub-studies (S1, S2, S3; *n* = 540 measurements total) varying simultaneous tag count, inter-tag separation, and angular configuration in 0.9% saline.

The multi-tag data define a narrower envelope than single-tag operation. In air at coaxial alignment and 20 mm separation, one to three simultaneous tags were identified in 100% of trials; performance declined for larger tag counts. For *N* = 3 at coaxial alignment, separation ≥ 20 mm also produced 100% success. In 0.9% NaCl at *N* = 3 and Δ = 20 mm, however, success fell from 100% at 0° to 56.7% at 30° and 0% at 60° and 90°. The validated 100% multi-tag envelope is therefore restricted to the tested coaxial condition; angular misalignment is a major limitation. Full per-condition results are shown in Supplementary Tables S9–S11.

**Kalman-filter distance estimation validation**

The dynamic-estimator dataset contains 1950 samples acquired/resampled at 50 Hz across four predefined trajectories. It was used to compare the as-shipped Kalman tuning, a re-tuned configuration, and a moving-average baseline. This analysis is secondary to the binary detection endpoint and is reported in detail in Supplementary Section S10.

The as-shipped Kalman tuning produced substantial lag on dynamic trajectories, while the re-tuned configuration reduced RMSE on the tested trajectories. Because the 50 Hz estimator validation is an offline/secondary analysis and depends on the empirical RSSI-to-distance mapping, the main conclusion is limited to firmware-development guidance rather than a claim of validated surgical localisation accuracy.

**Validation status of the Must-Have requirements set**

The present study experimentally verifies the coaxial reference medium range, within-device success proportion, response time, battery endurance, and shaft-compatible subassembly dimensions. The measured passive resonance and loaded Q characterise the antenna network, but formal 13.56 MHz carrier-frequency compliance, integrated-shaft electromagnetic performance, EMC/human-exposure qualification, biocompatibility, and sterilisation remain pending. Table 1 distinguishes these evidence levels explicitly.

### 3.8. Multi-Layer Phantom Detection Performance

The bench-top work was extended to five-layer tissue-equivalent phantoms to test how anatomical layering, perirectal-fat thickness, tag angle, and temperature affect detection. The primary phantom endpoints are detection probability and detection distance conditional on successful detection. The 37 °C design is explicitly unbalanced because non-coaxial measurements were available only from batch B1.

#### 3.8.1. Phantom Dielectric Verification

The dielectric characterisation of all three phantom batches (B1, B2, B3) confirmed quantitative agreement with the IT’IS Foundation database 4.1 targets at 13.56 MHz (Table 9, Figure S6 (Supplementary)). Mean deviations from the IT’IS reference values were +0.7% to +1.4% in permittivity and +0.7% to +1.3% in conductivity across all five strata, all within the ±10% tolerance criterion adopted for tissue-equivalent phantoms in the biomedical electromagnetic sensing literature [39]. Inter-batch coefficients of variation remained below 2.5% for all strata, demonstrating reproducible fabrication. Temporal stability monitoring across the 30 min–4 h post-fabrication window confirmed dielectric drift below 5% (mean drift across all strata: 1.6%), validating the 4-h operational window for detection measurements.

#### 3.8.2. Detection Feasibility Across the Multi-Layer Configuration

At 22 °C, detection succeeded in 100% of trials for fat = 0 mm at 0–60° and for fat = 10 mm at 0°. At fat = 0 mm and 90°, success was 86.7%. With 10 mm fat, success fell to 18.9% at 30° and to 0% at 60° and 90°. No detections occurred at fat thicknesses of 20 or 30 mm in any tested angle. These results define a conditional laboratory operating envelope: the ≥15 mm range target is met under coaxial conditions, but reliable detection is not maintained across all angular/tissue configurations.

#### 3.8.3. Detection Range and Per-Cell Statistics

Detection distances are reported only among successful detections (Table 10), while success proportions are treated separately. Coaxial mean ranges at 22 °C were 16.23 ± 0.28 mm with 0 mm fat and 16.16 ± 0.30 mm with 10 mm fat. The close agreement of the 0 mm fat coaxial value with saline should be interpreted as configuration-specific; the strong loss of detection probability at 10 mm fat and non-coaxial angles demonstrates that layered tissue performance cannot be inferred from saline attenuation alone.

#### 3.8.4. FEM Cross-Validation

For the six detectable configurations compared with FEM, Pearson r^2^ was 0.994, RMSE was 0.81 mm, MAE was 0.70 mm, and mean FEM–experiment bias was +0.70 mm. The 95% limits of agreement were [−0.18, +1.59] mm [40]. These absolute-error metrics are reported alongside correlation because correlation alone does not establish agreement; the per-configuration values are reported in Table 11.

The FEM model predicted feasibility in several configurations in which the experiment produced no detections, particularly at larger fat thickness and/or unfavourable angles. This discrepancy indicates that the present FEM geometry does not capture all interface, spacing, or coupling effects, and limits its use for extrapolating the detection boundary. Model refinement is therefore required before predictive use outside the experimentally validated region.

#### 3.8.5. Temperature Sensitivity Assessment (22 °C vs. 37 °C)

Across the detectable configurations measured at both temperatures, mean range differences were small, and no FDR-corrected per-cell comparison reached statistical significance. However, the 37 °C dataset is unbalanced; non-coaxial cells were measured only in batch B1, whereas the 22 °C dataset includes three batches. The TOST result is therefore treated as exploratory and does not establish batch-independent thermal equivalence across the full configuration matrix. Supplementary Section S16.3 states this limitation explicitly.

**Figure 8:**
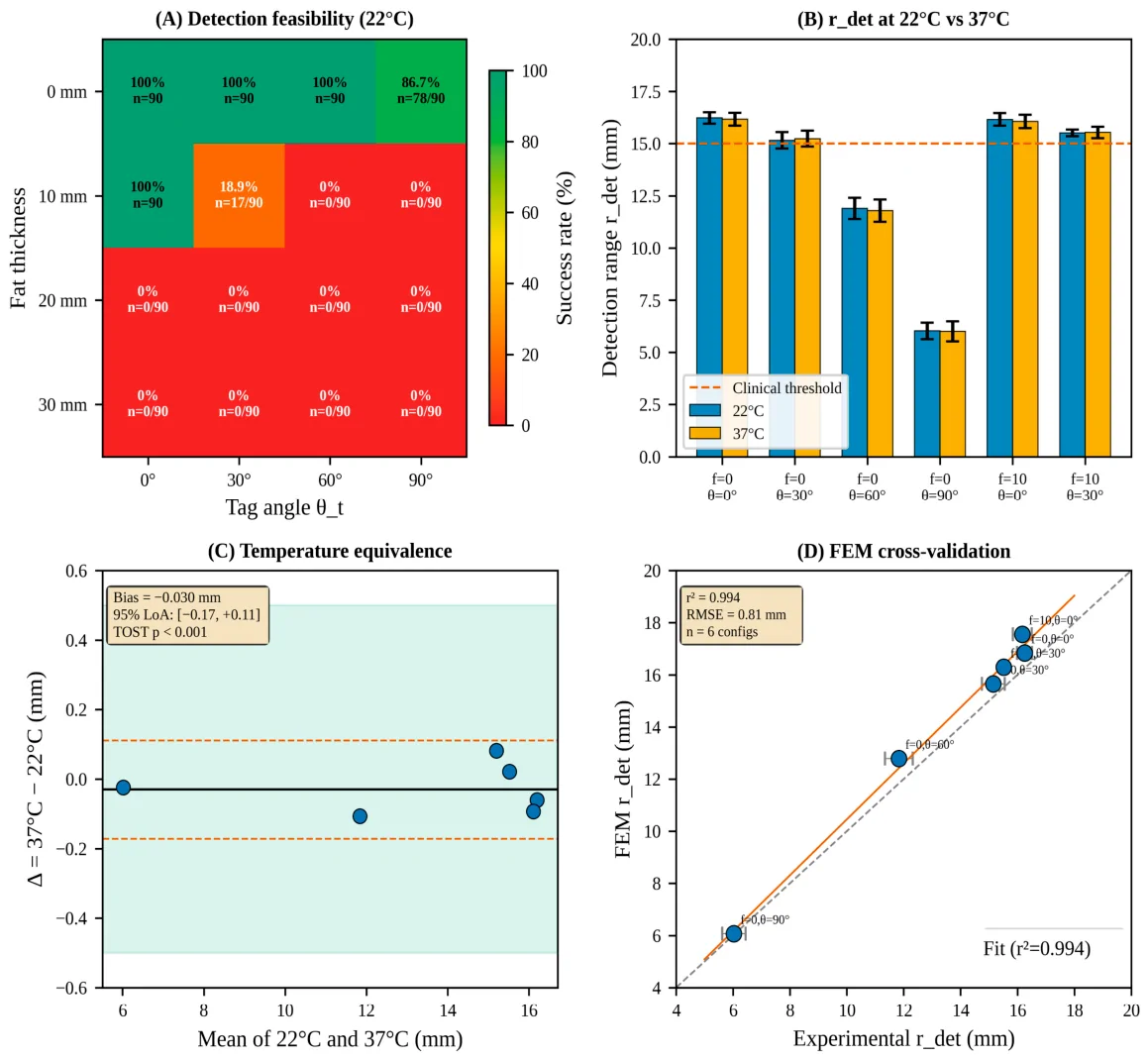
Multi-layer phantom results. (**A**) Detection success map at 22 °C across fat thickness and tag angle. (**B**) Detection distance among successful trials at 22 °C and 37 °C. (**C**) Temperature-difference analysis; because the 37 °C non-coaxial subset contains only batch B1, this panel is interpreted as exploratory rather than as full batch-independent equivalence evidence. (**D**) FEM–experiment comparison across six detectable configurations (r2 = 0.994, RMSE = 0.81 mm, bias +0.70 mm). In panel (**B**), blue and gold bars denote 22 °C and 37 °C means, respectively, with error bars showing dispersion and the orange dashed line indicating the 15 mm reference threshold. In panel (**C**), circles denote configuration-specific temperature differences, the solid horizontal line denotes mean bias, and dashed horizontal lines denote the 95% limits of agreement. In panel (**D**), circles denote configuration pairs, the grey dashed line is the identity line, and the orange line is the fitted regression.

Within the available data, temperature effects appear small relative to the dominant angular and geometric effects, but confirmation in a balanced multi-batch design remains necessary. The combined phantom results are summarised in Figure 8.

#### 3.8.6. Inter-Batch Reproducibility

Across the three 22 °C phantom batches, inter-batch coefficients of variation in fully detectable cells remained low (<1.6%), although several cell-wise tests detected statistically significant batch effects. With only three batches, the between-batch variance estimate remains imprecise and should not be overinterpreted. The GUM budget therefore retains inter-batch variability as an explicit contributor (Table 12), and the ex vivo phase will expand the number of independent specimens/prototypes.

## 4. Discussion

### 4.1. Achievement of Design Objectives

The principal experimentally demonstrated achievements are shaft-compatible antenna/electronics geometry, close agreement between analytical and measured inductance, measured loaded Q = 23, coaxial detection range > 15 mm in the air/saline reference tests, within-device detection consistency, real-time response, measured battery endurance, and a quantified multi-layer phantom envelope. These achievements do not imply that every Must-Have programme requirement is fully validated; integrated-shaft RF performance, formal carrier frequency compliance, biocompatibility, sterilisation, EMC, and human-exposure qualification remain pending.

The central methodological contribution is better described as a physics-informed design plus experimentally anchored detection modelling. Geometry/material calculations and FEM provide an independent basis for the antenna field profile, whereas the final field-to-detection mapping uses empirical anchoring. This separation clarifies the evidential role of each model component, and avoids conflating calibration consistency with independent validation.

The bench-top GUM result (U = 0.63 mm, k = 2) is a hybrid estimate because it includes a model-based inter-operator term. It characterises the distance-threshold measurement process for the present setup, and should not be interpreted as sub-millimetre 3D localisation accuracy or as multi-operator reproducibility. The dominant experimental result remains the strong angular dependence (partial η^2^ = 0.989), which directly motivates probe-orientation strategies and future multi-axis antenna concepts.

The Kalman-filter study is retained as a secondary firmware development result. The as-shipped conservative tuning exhibited dynamic lag, and re-tuning improved RMSE on the predefined trajectories. Because the analysis uses an empirical RSSI-to-distance conversion and a separate 50 Hz dataset, it is not used to claim validated intraoperative localisation accuracy.

The multi-layer phantom campaign is the most clinically relevant evidence in the present study because it exposes the limitations hidden by the air/saline comparison. Reliable detection was maintained with 10 mm fat only at coaxial alignment; at the same thickness, success fell to 18.9% at 30° and to zero at 60° and 90°. No detections occurred at the 20–30 mm fat level. The design requirement should therefore be understood as conditional on geometry and orientation, and systematic probe rotation or a multi-axis field geometry is likely necessary for broader anatomical coverage.

The available 22 °C/37 °C comparisons suggest that temperature contributes less variability than angle or fat thickness, but the unbalanced batch structure prevents a strong batch-independent equivalence claim. A balanced temperature study should accompany the next multi-prototype or ex vivo campaign.

The FEM cross-validation (r^2^ = 0.994, 95% CI [0.943, 0.999] across *n* = 6 detectable configurations) exposed a systematic FEM overestimation (bias +0.70 mm, RMSE 0.81 mm) that grows with tissue-path complexity—from +0.04 mm at fat = 0 mm, θ = 90° to +1.40 mm at fat = 10 mm, θ = 0°. The homogeneous fat layer FEM idealisation predicted detection feasibility at all fat ≥ 10 mm angular configurations and at fat = 20–30 mm, whereas the experiment yielded failure beyond fat = 10 mm, θ = 0°. This divergence is attributed to air gap transmission losses at the phantom–antenna interface and impedance mismatch boundary effects not captured in the present FEM model. The finding identifies model refinement (incorporation of layer-interface boundary conditions and explicit air-gap modelling) as a specific target for the next design iteration.

### 4.2. Physics-Informed Electromagnetic Design and Model Consistency

The analytical workflow combines finite-rod demagnetisation, inductance/resonance calculations, and an axial-field model with FEM cross-validation. Its strongest independent validation is the agreement between calculated and measured antenna quantities, particularly L_ant = 17.4 µH (predicted) versus 16.9 µH (measured), together with the 4.2% RMS analytical–FEM field profile agreement. These results support the usefulness of the design framework for constrained ferrite-rod antenna development.

At the detection threshold level, H_det_ and η_⊥_ are empirically anchored, while H_ref_ = 3.0 A/m is literature-informed rather than directly measured for the specific tag. The compact field margin across the measured configurations is therefore an internal consistency result after calibration. It indicates that the model shape is compatible with the data, but does not constitute a held-out prediction. A predefined calibration/validation split using the raw dataset would provide the appropriate next test of predictive performance.

This separation preserves the transferability of the geometry- and material-based antenna model while recognising that tag- and reader-specific detection parameters require empirical calibration followed by independent validation.

### 4.3. Comparison with the Preceding Tri-Frequency Reference and Inductive-Sensing Alternatives

Compared with the larger Ø5 × 15 mm reference antenna of [21], the present Ø3 × 25 mm configuration trades detection range for dimensional compatibility. Saline coaxial range decreases from 20.7 ± 1.2 mm to 16.26 ± 0.21 mm, while antenna diameter decreases by 40%. The present study adds the direct characterisation of the fabricated antenna, angular response, multi-layer phantom performance, and absolute FEM–experiment error metrics; the principal comparison is summarised in Table 13. Analytical unloaded-Q estimates are not used as evidence of performance improvement; the measured loaded Q = 23 is the relevant operational quantity.

The small air–saline range difference at coaxial alignment is useful for cross-study comparison, but does not establish negligible tissue attenuation in general. The phantom data demonstrate that geometry and orientation can dominate detection success even when coaxial saline and phantom ranges are similar. Consequently, the saline experiment is retained as a conductive reference condition rather than as a conservative predictor of ex vivo performance.

Set against the inductive proximity-sensing approaches of Calborean et al. and Bințințan et al. [17,18,19], the picture is instructive, as they achieved 15–25 mm through-tissue ranges for metallic markers, comparable to the present 16.26 mm. What separates them is marker capability; inductive sensing responds to any conductive object, whereas RFID resolves a unique identifier through ISO 14443-A. For multi-marker resection-margin protocols in total mesorectal excision [41], where proximal, distal, and satellite lesion tags must be discriminated, unique identification is a categorical prerequisite, not a marginal advantage. The two pathways therefore complement rather than compete. A parallel RFID-integrated endoscopic-clip approach for laparoscopic marking has also been reported [20], illustrating the broader interest in RFID-based marker integration complementary to the present implantable transponder approach.

### 4.4. Clinical Translation Context

The clinical motivation remains the need for the reliable localisation of small or poorly palpable colorectal lesions during minimally invasive surgery. Existing tattooing, clip-based, radioguided, and wireless marker approaches provide relevant precedents [8,9,10,11,12,13,14,15,16,17,18,19,20], but the present study does not estimate population-level clinical benefit or avoided complications from bench-top data. Its contribution is to define the engineering performance and limitations that must be addressed before effectiveness or cost-effectiveness can be evaluated clinically.

The phantom results translate directly into design constraints rather than clinical-effectiveness claims. Coaxial detection remains reliable through the tested 10 mm fat layer, but modest angular misalignment markedly reduces success. This supports systematic probe-orientation control and motivates future multi-axis antenna geometries. The current temperature dataset suggests limited thermal sensitivity, but balanced multi-batch confirmation is required before eliminating temperature-related verification from later validation stages.

### 4.5. Multi-Tag Operating Envelope

Multi-tag testing shows that up to three tags separated by at least 20 mm can be discriminated with 100% success under the tested coaxial air condition. This result does not extend to 30° misalignment in saline, where success for *N* = 3 and Δ = 20 mm was 56.7%. Accordingly, the demonstrated multi-tag operating envelope is conditional on coaxial orientation, with improved angular robustness remaining a target for subsequent development and validation.

The observed separation and angle dependence are relevant for planning future marker configurations, but specific clinical placement schemes were not evaluated in patients or ex vivo tissue. The present data support engineering constraints on tag count, spacing, and orientation rather than a validated surgical marking protocol.

Potential mechanisms include mutual coupling among co-resonant tags and anti-collision protocol overhead. These mechanisms remain hypotheses within the present dataset because coil currents and per-tag loading were not independently measured. Firmware optimisation and sequential interrogation strategies can be evaluated in future work after the integrated-shaft and tissue-validation steps.

### 4.6. Limitations

Several limitations bound the interpretation of this study, and mark the distinction between laboratory TRL 4 validation and a clinically qualified system.

All primary detection measurements derive from one fabricated antenna–electronics assembly, one operator, one transponder manufacturing batch, and repeated observations across sessions. The large number of cycles increases precision for within-device estimates but does not substitute for independent prototype, operator, or tag-batch replication. The model-based pre-trial inter-operator term in the bench GUM budget therefore requires replacement by direct experimental measurements in the planned multi-operator validation campaign.

The fully assembled shaft/handle prototype is shown in Figure 4b, but primary electromagnetic characterisation was performed on the antenna–electronics subassembly outside the stainless-steel shaft. Integrated-shaft resonance, matching, Q, tuning range, detection distance, and angular response remain unverified. Accordingly, the bench measurements should be interpreted as an electromagnetic validation of the antenna–electronics subassembly, rather than of the fully integrated instrument.

Multi-tag measurements were obtained on the same prototype and show strong angular dependence; dynamic estimator testing used predefined trajectories and a separate 50 Hz dataset. Neither multi-tag performance through the layered phantom nor combined dynamic/multi-tag operation has been established. The firmware results therefore offer developmental rather than clinical performance evidence.

Biocompatibility, sterilisation, formal IEC 60601-1-2 EMC testing, and human-exposure assessment were not completed. IEC 62209-1 [42] covers a frequency range that does not include 13.56 MHz and is therefore not used as the basis for exposure assessment in this study. No numerical SAR claim is made. Formal exposure assessment for the integrated device should follow an applicable framework such as IEC 62311:2019 [43], together with relevant regional exposure limits.

The five-layer phantoms improve anatomical relevance but do not reproduce perfusion, bowel curvature/deformation, heterogeneous tumour microenvironment, surgical motion, surrounding instruments, or long-term tag–tissue interaction. Tag migration and rotation between endoscopic placement and surgery were not evaluated, but are particularly important because angular sensitivity is a dominant limitation. The 37 °C dataset is also unbalanced across batches. These factors require direct ex vivo/in vivo investigation.

### 4.7. Future Directions and Planned Development Roadmap

The next validation stage should prioritise issues that directly limit the interpretation of the present results, as follows: integrated-shaft electromagnetic testing; at least three independently fabricated prototypes; multiple operators and tag batches; balanced 22 °C/37 °C replication where temperature is studied; ex vivo porcine colorectal tissue with realistic curvature and fat; and the explicit measurement of tag migration/rotation. Regulatory testing for biocompatibility, sterilisation, EMC, ingress protection, and human exposure should follow once the integrated hardware configuration is frozen.

A multi-axis antenna configuration remains a plausible engineering response to the strong orientation dependence observed here, but its localisation performance is prospective. The model-based Cramér–Rao and Kalman analyses in the Supplementary Materials may inform that design; they are not evidence that the current single-axis prototype provides 3D localisation.

The phantom data identify two immediate design priorities: improving angular robustness and extending the reliable range under thicker fat layers. FEM refinement should incorporate experimentally relevant interfaces and geometry before it is used to extrapolate beyond detectable configurations.

## 5. Conclusions

This study presents a shaft-compatible 13.56 MHz NFC antenna–reader platform for laparoscopic colorectal tumour-localisation research, and evaluates it from the perspective of analytical antenna design through bench and multi-layer phantom testing. The Ø3 × 25 mm Material 67 antenna achieved measured inductance of 16.9 µH (versus a 17.4 µH analytical estimate) and an operational loaded Q of 23.

Agreement among the analytical, FEM, and measured antenna quantities provides strong support for the physics-informed design framework. The subsequent field-to-detection mapping remains empirically anchored through a system threshold and transverse coupling term, and is therefore interpreted as model consistency evidence rather than as a calibration-independent predictive validation.

Experimentally, the coaxial range reached 16.45 ± 0.29 mm in air and 16.26 ± 0.21 mm in saline, while angle was the dominant determinant of range. In the five-layer phantom, detection was reliable at 0 and 10 mm fat under coaxial alignment, but deteriorated sharply with misalignment and failed at fat thicknesses ≥ 20 mm. FEM agreement across detectable configurations was high (r^2^ = 0.994), with RMSE 0.81 mm and bias +0.70 mm. These results define a conditional operating envelope rather than a general through-tissue performance guarantee.

Multi-tag discrimination was 100% for up to three tags separated by ≥20 mm under coaxial conditions, but fell substantially with angular misalignment. The bench-top GUM value U = 0.63 mm is a hybrid within-setup estimate because its inter-operator term is model-based, and the multi-layer phantom uncertainty budget is reported separately in the Supplementary Materials.

The present evidence supports the TRL 4 laboratory validation of the antenna–electronics sensing concept, and justifies progression to ex vivo testing. Before clinical claims would be appropriate, the complete shaft-integrated instrument must be electromagnetically verified across multiple prototypes/operators, tag stability and orientation must be characterised, and formal safety, EMC, biocompatibility, sterilisation, and human-exposure assessments must be completed.

The multi-layer phantom results identify the principal priorities for the next design iteration, namely, improved angular robustness, extended performance under thicker fat layers, interface-aware FEM modelling, and balanced multi-batch validation.

## Figures and Tables

**Table 1:** Key IntelliSense design requirements with quantitative specifications, priorities, and current validation status.

Requirement	Specification	Priority	Validation Status
Detection range, coaxial reference condition	≥15 mm	Must	Conditionally validated: 16.26 mm in saline at 0°; not maintained across all phantom angles/thicknesses
Detection success rate (*n* = 60 per condition)	≥95%	Must	Experimentally validated (98.5%)
Response time	<100 ms	Must	Experimentally validated (45 ± 8 ms)
Battery autonomy	>4 h	Must	Experimentally validated (9.5 h at 50% duty cycle)
Shaft diameter	≤12 mm	Must	Geometrically verified (PCB and antenna assembly within envelope)
Working length	300–400 mm	Should	Geometrically verified (350 mm shaft length, design target configuration)
Operating frequency	13.56 MHz ± 7 kHz	Must	Reader configured for 13.56 MHz; passive network resonance measured separately; formal carrier frequency compliance verification pending
Biocompatibility	ISO 10993 series [24]	Must	Planned regulatory activity (ex vivo phase)
Sterilisation	Autoclave/EtO compatible	Must	Planned regulatory activity (ex vivo phase)
Electromagnetic safety/exposure	IEC 60601-1-2 [25]; applicable human-exposure assessment	Must	Preliminary characterisation only; formal EMC and human-exposure qualification planned

**Table 2:** Geometry- and model-derived antenna parameters of the shaft-compatible Ø3 × 25 mm Material 67 configuration compared with the Ø5 × 15 mm reference of Mocan et al. [21]. Analytical estimates and inferred reference values are identified explicitly.

Parameter	Symbol	Present Work	[21] Reference	Ratio
Ferrite material	—	Material 67	NiZn (μ_i = 125)	—
Rod dimensions	L × D	25 × 3 mm	15 × 5 mm	—
Aspect ratio	L/D	8.33	3.0	2.78×
Demagnetisation factor	N_z	0.027	≈0.10	0.27×
Effective permeability	μ_eff	19.58	≈10.7	1.83×
Coil turns	*N*	20	10	2.0×
Antenna inductance	L_ant	17.4 µH	≈0.8 µH	21.8×
Resonance capacitance	C_res	7.9 pF	≈145 pF (estimated)	0.054×
AC winding resistance (idealised)	R_AC	343 mΩ (analytical estimate)	*n*/a	—
Ferrite loss contribution	R_ferrite	model-dependent estimate	*n*/a	—
Total loss resistance	R_tot	idealised analytical estimate	*n*/a	—
Unloaded quality factor	Q_unloaded	≈3700 (idealised upper-bound estimate)	≈50–100 (estimated)	not directly comparable
Loaded quality factor	Q_loaded	23 measured; 20–80 design range	30–80 (estimated)	operational comparison
Equivalent magnetic moment	m	0.277 mA·m^2^	*n*/a (different coil)	—

**Table 3:** Measured antenna and matching parameters versus analytical design targets from Section 2.4.

Parameter	Symbol	Derived (Section 2.4)	Measured	Deviation
Antenna inductance	L_ant	17.4 µH	16.9 µH	−2.9%
Resonance capacitance	C_res	7.9 pF	8.2 pF (E12 selection)	+3.8%
Passive matched-network resonance (pre-AAT)	f_0_	13.56 MHz target	13.58 MHz	+0.15%; not a carrier frequency compliance test
Return loss at f_0_	S_11_	<−20 dB	−24 dB	Exceeds spec
−3 dB bandwidth	BW	~600 kHz	580 kHz	−3.3%
Loaded quality factor	Q_loaded	20–80	23	within range

**Table 4:** Detection-distance performance across the 8-condition experimental matrix (*n* = 30 measurement repetitions per encapsulation variant per condition; 60 measurements aggregated per condition row).

Medium	Angle (°)	*n*	Mean (mm)	SD (mm)	95% Bias-Corrected and Accelerated (BCa) CI (mm)	Shapiro *p*	Normality
Air	0	60	16.45	0.29	16.35–16.55	0.952	Normal
Air	30	60	14.64	0.38	14.50–14.77	0.581	Normal
Air	60	60	12.27	0.49	12.11–12.45	0.030	Non-normal
Air	90	60	5.92	0.52	5.74–6.12	0.088	Normal
0.9% NaCl	0	60	16.26	0.21	16.18–16.34	0.543	Normal
0.9% NaCl	30	60	14.63	0.39	14.50–14.78	0.954	Normal
0.9% NaCl	60	60	12.21	0.38	12.08–12.34	0.808	Normal
0.9% NaCl	90	60	5.76	0.55	5.57–5.96	0.582	Normal

**Table 5:** Mixed-effects two-way ANOVA on the 480-measurement primary dataset (240 per encapsulation × 2 variants; medium × angle main effects, encapsulation as covariate, session as random factor; *n* = 60 per medium × angle cell, residual df reflecting Satterthwaite approximation under mixed-effects estimation).

Source	df	F	*p*	η^2^_partial
Angle	3	7399.2	≪0.001	0.989
Medium	1	3.95	0.048	0.017
Angle × Medium	3	0.68	0.565	0.009
Residual	232	—	—	—

**Table 6:** Physics-informed field-to-detection consistency analysis. H_axial is calculated from the analytical field model and H_coupled includes the empirically anchored transverse-coupling term. H_ref = 3.0 A/m is used only as a literature-informed reference. The table is a calibration/consistency analysis and not a fully independent ab initio validation of the specific tag activation threshold.

Medium	Angle (°)	r_det (mm)	H_axial (A/m)	H_coupled (A/m)	Reference Ratio (×H_ref)
Air	0	16.45	5.00	5.00	1.67
Air	30	14.64	6.18	5.43	1.81
Air	60	12.27	8.20	4.62	1.54
Air	90	5.92	16.66	5.00	1.67
0.9% NaCl	0	16.26	5.11	5.11	1.70
0.9% NaCl	30	14.63	6.19	5.44	1.81
0.9% NaCl	60	12.21	8.26	4.66	1.55
0.9% NaCl	90	5.76	16.91	5.07	1.69

**Table 7:** Effective RSSI–distance exponent estimates from the empirical dense-sampling sub-dataset. Values are descriptive over the stated fitting interval and are not used as independent validation of the analytical field model.

Medium	Angle (°)	n_eff	A (dB)	R^2^	Residual (%)
Air	0	4.008	48.61	0.998	+0.20
Air	30	4.003	46.97	0.999	+0.07
Air	60	3.998	43.45	0.998	−0.05
Air	90	4.003	30.72	0.998	+0.08
0.9% NaCl	0	3.978	51.37	0.997	−0.55
0.9% NaCl	30	3.947	48.94	0.999	−1.33
0.9% NaCl	60	3.996	45.58	0.998	−0.09
0.9% NaCl	90	3.994	31.24	0.997	−0.14
Mean ± SD	—	3.99 ± 0.02	—	—	—

**Table 8:** Hybrid GUM uncertainty budget for bench-top detection distance measurement. A * denotes a model-based inter-operator term that remains to be replaced by direct multi-operator data.

Source	Type	u_i (mm)	u_i^2^ (mm^2^)	Notes
Repeatability (within-condition SD at 0°)	A	0.220	0.048	Measured within-prototype repeatability
Inter-operator reproducibility	A *	0.180	0.032	Model-based pre-trial estimate
Goniometer angular resolution (0.5°)	B	0.090	0.008	Rectangular, ×∂d/∂θ
XYZ stage calibration	B	0.050	0.003	Certificate, k = 2
Temperature variation (±2 °C)	B	0.046	0.002	Rectangular
Tag manufacturing tolerance	B	0.060	0.004	Rectangular
Electronic noise (ST25R3916B)	B	0.035	0.001	Rectangular
Combined standard uncertainty	—	0.31	0.098	u_c = √Σu_i^2^
Expanded uncertainty (k = 2)	—	0.63	—	U = k · u_c, 95% confidence

**Table 9:** Phantom dielectric properties at 13.56 MHz: measured (mean ± SD across 3 batches × 4 timepoints × 15 replicates, *n* = 180 per layer) versus IT’IS Foundation database 4.1 reference values.

Layer	ε_r Measured	ε_r IT’IS	Δε_r	σ Measured [S/m]	σ IT’IS [S/m]	Δσ
Mucosa	85.9 ± 1.5	85.0	+1.1%	1.007 ± 0.029	1.000	+0.7%
Submucosa *	65.5 ± 1.3	65.0	+0.7%	0.808 ± 0.022	0.800	+1.0%
Muscularis prop.	58.6 ± 1.0	58.0	+1.1%	0.608 ± 0.014	0.600	+1.3%
Serosa	52.4 ± 1.1	52.0	+0.8%	0.504 ± 0.013	0.500	+0.8%
Perirectal fat	5.58 ± 0.12	5.50	+1.4%	0.0405 ± 0.0013	0.040	+1.3%

* Submucosa = transponder implantation layer. All Δ values within ±10% IT’IS-compliance tolerance for tissue-equivalent phantoms [39].

**Table 10:** Detection range (mean ± SD) across the multi-layer phantom configuration matrix at 22 °C and 37 °C. Per-cell sample sizes (*n*_succ/*n*_total) explicit.

Fat [mm]	Tag Angle	r_det @ 22 °C [mm]	*n*_succ/*n*_tot @ 22 °C	r_det @ 37 °C [mm]	*n*_succ/*n*_tot @ 37 °C
0	0°	16.23 ± 0.28	90/90 (100%)	16.17 ± 0.31	90/90 (100%)
0	30°	15.16 ± 0.39	90/90 (100%)	15.24 ± 0.38	30/30 (100%) *
0	60°	11.90 ± 0.51	90/90 (100%)	11.79 ± 0.53	30/30 (100%) *
0	90°	6.03 ± 0.40	78/90 (86.7%)	6.00 ± 0.48	27/30 (90.0%) *
10	0°	16.16 ± 0.30	90/90 (100%)	16.06 ± 0.32	89/90 (98.9%)
10	30°	15.51 ± 0.16	17/90 (18.9%)	15.54 ± 0.27	7/30 (23.3%) *
10	60°	—	0/90 (0%)	—	0/30 (0%) *
10	90°	—	0/90 (0%)	—	0/30 (0%) *
20	All	—	0/360 (0%)	—	0/120 (0%)
30	All	—	0/360 (0%)	—	0/120 (0%)

* Reduced sample at 37 °C for non-coaxial cells: only batch B1 measured. Wilson 95% upper bound on success rate at fat = 10 mm, θ_t = 90° (combined *n* = 120): 3.10%.

**Table 11:** FEM experimental cross-validation across the multi-layer phantom DETECTABLE configurations (*n* = 6).

Configuration (Fat, Angle)	FEM r_det [mm]	Exp r_det [mm]	Δ [mm]	Δ [%]
0 mm, 0°	16.84	16.23 ± 0.28	+0.61	+3.8%
0 mm, 30°	15.65	15.16 ± 0.39	+0.49	+3.2%
0 mm, 60°	12.79	11.90 ± 0.51	+0.89	+7.5%
0 mm, 90°	6.07	6.03 ± 0.40	+0.04	+0.7%
10 mm, 0°	17.56	16.16 ± 0.30	+1.40	+8.7%
10 mm, 30°	16.29	15.51 ± 0.16	+0.78	+5.0%

Pearson r = 0.997 (Fisher-z 95% CI: [0.971, 1.000]); *r*^2^ = 0.994 (95% CI: [0.943, 0.999]); RMSE = 0.81 mm; MAE = 0.70 mm; Bland–Altman bias = +0.70 mm (95% CI: [+0.23, +1.18]); 95% LoA: [−0.18, +1.59] mm; proportional-bias regression slope = 0.076 (*p* = 0.121, no proportional bias). *n* = 6 detectable configurations.

**Table 12:** GUM uncertainty budget for multi-layer phantom detection distance measurements. Rectangular distribution source limits are converted to standard uncertainties before root-sum-of-squares propagation. DOF, degrees of freedom; ∞ denotes effectively infinite degrees of freedom for Type B components.

Contributor	Type	u_i [mm]	Distribution	DOF	Source	Contribution
XYZ stage positioning	B	0.058	Rectangular	∞	±0.1 mm half-width/√3	0.8%
Within-condition repeatability	A	0.330	Normal	29	Experimental pooled SD	26.6%
Phantom dielectric tolerance	B	0.416	Rectangular	∞	±10% × sensitivity, then/√3	42.3%
Inter-batch variability	B	0.182	Normal	2	Three-batch estimate	8.1%
Temperature ±0.5 °C	B	0.115	Rectangular	∞	Sensitivity-scaled half-width/√3	3.2%
RF/EMC noise	B	0.150	Normal	∞	Bench characterisation	5.5%
Tag positioning depth	B	0.202	Rectangular	∞	±0.2 mm × sensitivity, then/√3	10.0%
Goniometer ±1°	B	0.104	Rectangular	∞	Sensitivity-scaled half-width/√3	2.6%
Stage zero calibration	B	0.060	Normal	∞	Stage repeatability test	0.9%
Combined u_c (RSS)	—	0.640	—	—	Root-sum-of-squares	100%
Expanded U (k = 2)	—	±1.28	—	—	k = 2, approx. 95% coverage	—

**Table 13:** Comparative performance metrics: present IntelliSense laparoscopic-compatible configuration versus the preceding bench-top tri-frequency reference of Mocan et al. [21].

Metric	Present (Ø3 × 25, Material 67)	[21] Reference (Ø5 × 15, NiZn μ_i = 125)	Change
Detection range, air	16.45 ± 0.29 mm	23.0 ± 1.1 mm	−28.5%
Detection range, saline	16.26 ± 0.21 mm	20.7 ± 1.2 mm	−21.4%
Air-to-saline attenuation	1.2%	10.0%	−88%
Antenna diameter	3 mm	5 mm	−40%
Antenna aspect ratio L/D	8.33	3.0	+178%
Effective permeability μ_eff	19.58	10.7	+83%
Unloaded-Q analytical estimate	≈3700 (idealised upper bound)	≈50–100 (estimated)	not used for performance comparison
Shaft compatibility (≤12 mm)	Yes (11 mm envelope)	No	qualitative

## Data Availability

The datasets supporting the study are available from the corresponding author on reasonable request, subject to ongoing patent and pre-clinical-development constraints. Analysis scripts and the firmware source/configuration relevant to the reported measurements are likewise available on reasonable request where compatible with those intellectual property constraints.

## Supplementary Materials

The following supporting information can be downloaded at: https://www.mdpi.com/article/10.3390/s26185759/s1.
