# Supplementary material for: Physics-Informed Design and Bench/Phantom Validation of a Shaft-Compatible 13.56 MHz NFC System for Laparoscopic Colorectal Tumour Localisation
Source: Sensors (Basel). 2026 Sep 10;26(18):5759. doi: 10.3390/s26185759 (PMC13611037; doi:10.3390/s26185759)
Supplement: Supplementary file 1 [file sensors-26-05759-s001.zip › sensors-4427239-supplementary.pdf]

Supplementary Materials

Physics-Informed Design and Bench/Phantom Validation of a Shaft-Compatible 13.56 MHz NFC System for Laparoscopic Colorectal Tumour Localisation

S1. Requirements Engineering Documentation

This supplement provides the complete required engineering documentation for the IntelliSense system development, supporting reproducibility and regulatory traceability as referenced in Section 2.1 of the main manuscript. The documentation follows ISO 13485:2016 design control principles, IEC 62366-1:2015 usability engineering, and FDA Human Factors guidance for medical devices.

S1.1 Design Objective Statement

The overarching design objective for the IntelliSense system was formally articulated as follows:

> ***"To develop a mechatronic surgical instrument capable of real-time, non-ionising detection of miniaturised biocompatible transponders implanted in proximity to colorectal tumours, enabling precise intraoperative localisation during laparoscopic procedures with accuracy, reliability, and ergonomic characteristics suitable for integration into existing surgical workflows."***

S1.2 Requirements Traceability Matrix

Coverage analysis distinguishes implemented engineering traceability from programme-level needs that are not yet represented by a validated design input. In Table S1, UN-08 (non-ionising exposure), UN-10 (transponder positional stability), UN-13 (cost competitiveness), UN-14 (reimbursement), and UN-16 (clinical evidence) do not yet have an explicit DI linkage. These items therefore remain open programme-level requirements and are not counted as completed bidirectional traceability in the present prototype study.

Table S1. *Requirements Traceability Matrix for IntelliSense system development. Bidirectional linkage from user needs (UN-XX, Table S3) through design input specifications to verification methods and current validation status. Cross-references point to relevant sections of the main manuscript.*

DI-ID	Design Input Specification	Target Value	MoSCoW	Traces from	Verification Method	Validation Status & Cross-reference
DI-01	Coaxial detection range, reference medium	≥15 mm at θ = 0°	M	UN-01, UN-07	Bench testing in air/saline; phantom envelope characterised separately	Conditionally validated: 16.26 mm saline at 0°; not maintained across all phantom angles/thicknesses
DI-02	Within-device detection success	≥95% (n = 30 per condition)	M	UN-01	Per-condition aggregation across primary dataset	98.5% across primary repeated cycles; single-prototype result (§3.7)

DI-ID	Design Input Specification	Target Value	MoSCoW	Traces from	Verification Method	Validation Status & Cross-reference
	proportion					
DI-03	False positive rate (environmental NFC source rejection)	<1%	S	UN-01, UN-07	Bench testing with non-target NFC sources	Mitigated by UID verification (§2.6.1); formal characterisation pending
DI-04	Response time (detection latency)	<100 ms	M	UN-02	Oscilloscope-instrumented timing, n = 100	Validated 45 ± 8 ms (§3.7)
DI-05	RSSI update rate for proximity feedback	≥10 Hz	S	UN-02	Firmware timing measurement	Validated 22 Hz (45 ms cycle) (§2.2, §2.6.2)
DI-06	Shaft outer diameter	≤12 mm	M	UN-03	Dimensional measurement of integrated assembly	Geometrically verified: 11 mm antenna/electronics envelope and Ø12 mm mechanical shaft; primary RF characterisation was performed on the subassembly outside the shaft (§2.7.1; §4.6).
DI-07	Working length	300–400 mm	S	UN-03	Dimensional measurement	Validated 350 mm working length within 300–400 mm specification (§2.7.1, Table 1)
DI-08	Total instrument weight	<250 g	S	UN-03	Gravimetric measurement	Mechanically integrated prototype: 185 g including battery; RF-characterised antenna/electronics/battery subassembly: 78 g (§2.6.8; §2.7.1).
DI-09	Battery operating time at 50% duty cycle	>4 h	M	UN-04	Continuous-operation endurance, n = 3 trials	Validated 9.5 ± 0.3 h (§3.7)
DI-10	Power-on to operational state	<10 s	S	UN-04, UN-12	Boot-time measurement	Pending characterisation (§4.6)
DI-11	EMC immunity per IEC 60601-1-2	Pass all tests	M	UN-05	Accredited EMC test laboratory	Preliminary in-house characterisation only; formal accredited qualification pending
DI-12	Operation with electrosurgery active	No malfunction at >20 cm separation	S	UN-05	Interference testing with monopolar/bipolar units	Preliminary OK at >20 cm; formal pending (§4.6)
DI-13	Transponder biocompatibility	ISO 10993-series compliant	M	UN-06	ISO 10993 cytotoxicity, sensitisation, irritation panel	Glass NTAG216 has biocompatibility precedent (§2.3); device-level testing planned (§4.6, §4.7)
DI-14	Transponder envelope dimensions	≤Ø2.12 × 12 mm	S	UN-09	Dimensional measurement	Validated Ø2.12 × 12 mm NTAG216 glass capsule (§2.3)
DI-15	Sterilisation method compatibility	Autoclave or EtO	M	UN-11	ISO 17665 / EN 285 cycle validation	Materials selected for compatibility; formal validation deferred (§4.6, §4.7)

DI-ID	Design Input Specification	Target Value	MoSCoW	Traces from	Verification Method	Validation Status & Cross-reference
DI-16	Performance retention after sterilisation	<5% degradation @ 100 cycles	S	UN-11	Sterilisation-cycle endurance test	Planned regulatory activity (§4.6, §4.7)
DI-17	Electrical safety per IEC 60601-1	Type BF applied part	S	UN-15	Electrical safety testing at accredited laboratory	Subsumed under EMC/safety qualification programme; pending formal qualification (§4.6)
DI-18	Operating frequency compliance	13.56 MHz ± 7 kHz	M	UN-15	Reader configuration + VNA network characterisation; formal carrier spectrum test pending	Reader configured for 13.56 MHz; passive network resonance 13.58 MHz is not a carrier-frequency compliance measurement
DI-19	Multi-tag discrimination capability	N ≤ 3, Δ ≥ 20 mm, θ = 0°	S	UN-01	Multi-tag bench testing (S1, S2, S3, n = 540)	Validated at 100% only under coaxial condition; at 30° in saline success = 56.7% (§3.7; Tables S9–S11)

S1.3 Stakeholder Identification and Analysis

Comprehensive stakeholder analysis identified seven principal categories whose requirements must be addressed. Table S2 presents the stakeholder mapping with influence levels and primary concerns.

Table S2. *Stakeholder analysis for IntelliSense system development with influence classification and primary concerns.*

Stakeholder	Primary Interest	Influence	Key Concerns
**Colorectal Surgeons**	Clinical efficacy, workflow integration	High	Detection reliability, ergonomics, learning curve, procedure time impact
**Patients**	Safety, oncological outcomes	High	Biocompatibility, margin adequacy, complication rates, recovery
**Gastroenterologists**	Transponder deployment	Medium	Endoscopic implantation technique, tag visibility, deployment time
**OR Staff**	Workflow efficiency, safety	Medium	Sterilisation, setup time, storage, maintenance, EMC safety
**Hospital Administration**	Cost-effectiveness, liability	Medium	Acquisition cost, disposable costs, reimbursement, ROI
**Regulatory Authorities**	Safety, efficacy evidence	High	Clinical validation, risk management, standards compliance
**Biomedical Engineers**	Technical maintenance	Low	Serviceability, calibration, troubleshooting, spare parts

S1.4 Requirements Elicitation Process

Requirements were elicited through a multi-modal approach: (a) structured interviews with 5 colorectal surgeons (experience 8–25 years, >100 laparoscopic procedures annually), 3 gastroenterologists, and 4 OR nurses from two tertiary referral centres; (b) direct observation of 12 laparoscopic colorectal procedures (6 right hemicolectomies, 4 low anterior resections, 2 abdominoperineal resections); (c) systematic review of 47 publications on intraoperative localisation limitations; (d) regulatory analysis of FDA guidance, EU MDR requirements, and predicate device specifications (LOCalizer, SAVI SCOUT); (e) electromagnetic characterisation from our preceding frequency selection study [21].

S2. User Needs Specification

User needs represent the highest-level expression of stakeholder expectations, articulated in clinical and operational language. Table S3 presents the consolidated 16 user needs with MoSCoW classification.

Table S3. *User needs specification derived from stakeholder requirements elicitation (n = 16 needs).*

ID	Stakeholder	User Need Statement	Priority	MoSCoW
**UN-01**	Surgeon	The system shall reliably detect transponders through intact bowel wall tissue	Essential	M
**UN-02**	Surgeon	The system shall provide real-time feedback enabling progressive approach to target	Essential	M
**UN-03**	Surgeon	The instrument shall be ergonomically compatible with laparoscopic technique	Essential	M
**UN-04**	Surgeon	The system shall not significantly extend operative time	Important	S
**UN-05**	Surgeon	The system shall function without interference from electrosurgical devices	Essential	M
**UN-06**	Patient	The transponder shall be biocompatible and cause no adverse tissue reactions	Essential	M
**UN-07**	Patient	The system shall enable adequate surgical margins reducing recurrence risk	Essential	M
**UN-08**	Patient	The technology shall not expose patient to ionising radiation	Essential	M
**UN-09**	Gastro.	Transponder deployment shall be achievable via standard colonoscope	Essential	M
**UN-10**	Gastro.	Transponder position shall remain stable until surgical procedure	Essential	M
**UN-11**	OR Staff	The instrument shall be sterilisable using standard hospital protocols	Essential	M
**UN-12**	OR Staff	Setup and preparation time shall be minimal (<5 minutes)	Important	S
**UN-13**	Hospital	System cost shall be competitive with existing localisation methods	Important	S
**UN-14**	Hospital	The technology shall support reimbursement through existing billing codes	Desirable	C
**UN-15**	Regulatory	The system shall comply with applicable medical device standards	Essential	M
**UN-**	Regulatory	Clinical evidence shall demonstrate safety and efficacy	Essential	M

ID	Stakeholder	User Need Statement	Priority	MoSCoW
**16**				

S3. Design Input Specifications

The 19 design inputs in Table S1 cover the engineering and regulatory targets used for the present development stage. Nine are classified as Must-Have programme requirements and ten as Should-Have. Their evidential status is not uniform; some are measured on the antenna–electronics subassembly, some are geometric/design targets, and others remain planned regulatory activities. DI-19 (multi-tag discrimination) is a Should-Have requirement and is validated only for the explicitly stated coaxial operating condition. UN-10 (tag positional stability) remains open because the present study does not provide longitudinal migration/rotation data.

S4. Requirements Prioritisation

Requirements were prioritised using a modified MoSCoW framework adapted for medical-device development. Multi-tag discrimination (DI-19) is classified as a Should-Have requirement; the Could-Have category is reserved for wireless data-link and RSSI-display functions.

Table S4. *Requirements prioritisation using MoSCoW framework for medical device development.*

Priority	Requirements (DI-XX)	Count
**Must Have**	DI-01, DI-02, DI-04, DI-06, DI-09, DI-11, DI-13, DI-15, DI-18	9
**Should Have**	DI-03, DI-05, DI-07, DI-08, DI-10, DI-12, DI-14, DI-16, DI-17, DI-19	10
**Could Have**	Wireless data link, RSSI display	2
**Won't Have**	3D position tracking, robotic integration, cloud connectivity	3

S5. Design Constraints and Boundary Conditions

The design solution space is bounded by physical, regulatory, and clinical context constraints. Reproducible bench reference media are distinguished from actual layered tissue, and saline is not assumed to provide a conservative performance bound for all tissue geometries.

Table S5. *Physical constraints bounding the IntelliSense design solution space.*

Constraint	Description	Source
**Near-field range**	Detection range limited by r^{-3} magnetic field decay	EM theory
Conductive-medium loading	Air/saline difference is configuration-specific; layered geometry and angle can dominate performance	Present study / [21]
**Angular null zone**	Strong range reduction at orthogonal orientation; magnitude depends on geometry and medium	Frequency study [21]

**Ferrite permeability**	Effective μeff << μr due to demagnetisation (N ≈ 0.12)	Material physics
**Antenna Q–bandwidth**	Trade-off: high Q → range but narrow bandwidth	RF engineering

Table S6. *Regulatory constraints derived from applicable medical device standards.*

Constraint	Description	Standard/Guidance
**ISM band compliance**	Operating frequency 13.56 MHz ± 7 kHz	ITU Radio Regulations
**RF emission limits**	Radiated emissions within CISPR 11 Class B limits	IEC 60601-1-2
**Electrical safety**	Patient leakage current < 100 μA (Type BF)	IEC 60601-1
**Biocompatibility**	Implant materials per ISO 10993 series testing	ISO 10993-1:2018
**Usability engineering**	Risk analysis for use errors, user interface design	IEC 62366-1:2015
**Risk management**	Hazard analysis, risk controls, residual risk assessment	ISO 14971:2019
Human exposure assessment	Formal EMF exposure assessment for integrated device before clinical use	IEC 62311:2019 (where applicable) + regional limits

Table S7. *Clinical context constraints derived from surgical workflow and anatomical considerations.*

Constraint	Description	Source
**Trocar compatibility**	Shaft must pass through 12.5 mm standard trocar	Laparoscopic instruments
**Tissue thickness range**	Bowel wall 3–8 mm + perirectal fat 0–30 mm	Anatomical studies
**Procedure duration**	Typical procedure 2–4 hours; localisation phase <15 min	Clinical observation
**Sterilisation protocol**	Autoclave 134°C/18 min or EtO 37°C/12 h	Hospital CSSD
**Tag dwell time**	Transponder implanted 1–14 days before surgery	Clinical workflow

S6. Consolidated Design Specifications

Table S8 presents the target design specifications that governed all engineering development, with traceability to constraint sources and prioritisation.

Table S8. *Consolidated design specifications for IntelliSense system development.*

Parameter	Target Specification	Constraint Source	Priority
Detection Performance			
Coaxial detection range (reference medium)	≥15 mm at θ = 0°	Anatomy	Must
Detection success rate	≥95%	Clinical	Must
Response time	<100 ms	Workflow	Must
Operating frequency	13.56 MHz ± 7 kHz	Regulatory	Must
Dimensional Constraints			
Shaft diameter	≤12 mm	Trocar	Must
Working length	300–400 mm	Anatomy	Should

Instrument weight	<250 g	Ergonomics	Should
Transponder size	≤Ø2.12 × 12 mm	Endoscope	Should
Operational Requirements			
Battery autonomy	>4 hours @ 50% duty	Procedure	Must
Start-up time	<10 seconds	Workflow	Should
Sterilisation compatibility	Autoclave or EtO	Hospital	Must
Safety & Compliance			
Electrical safety classification	Type BF applied part	IEC 60601-1	Should
EMC compliance	IEC 60601-1-2 formal test pending	Regulatory	Must
Biocompatibility (transponder)	ISO 10993 series	Regulatory	Must

S7. Requirements Traceability Matrix

The RTM remains useful as a design-control structure linking user needs, design inputs, design outputs, and verification evidence. Quantitative coverage is evaluated against Table S1 rather than reported as blanket 100% traceability. In particular, tag migration/rotation stability (UN-10) is identified as an unresolved requirement for the ex vivo/in vivo programme.

S8. Multi-Tag Discrimination Raw Data Summary

This section provides the per-condition summaries for the three multi-tag studies (S1–S3; 540 interrogation cycles in total). Interpretation is conditional: S1 and S2 establish the tag-count and spacing envelope under coaxial air conditions, whereas S3 demonstrates that angular misalignment in saline sharply reduces multi-tag success. Signal/noise/score quantities remain instrument-specific receiver-channel units and should not be interpreted as calibrated field amplitudes.

Three observations follow directly from Tables S9–S11. (i) Under coaxial alignment, 1–3 tags at 20 mm separation achieved 100% success, and N = 3 achieved 100% success for separations ≥20 mm. (ii) Performance declined for larger tag counts and for separations below 20 mm. (iii) At N = 3, Δ = 20 mm in 0.9% NaCl, success fell from 100% at 0° to 56.7% at 30° and 0% at 60° and 90°. Accordingly, the validated 100% multi-tag envelope is restricted to coaxial alignment. The present data do not independently separate mutual-coupling effects from anti-collision protocol overhead.

Table S9. *Multi-tag study S1: per-condition raw data summary (n = 210; success rate vs. number of simultaneous tags N at fixed inter-tag separation Δ = 20 mm, coaxial alignment, air; 30 repetitions per condition).*

Condition	n	k	Success	95% Wilson CI	Signal (mean ± SD)	Noise (mean ± SD)	Score (mean ± SD)	Time, ms (mean ± SD)
1 tags	30	30	100.0 %	[88.6, 100.0]	0.200 ± 0.003	-0.0020 ± 0.0078	0.197 ± 0.010	125 ± 5
2 tags	30	30	100.0 %	[88.6, 100.0]	0.174 ± 0.003	-0.0004 ± 0.0130	0.170 ± 0.014	150 ± 5
3 tags	30	30	100.0 %	[88.6, 100.0]	0.154 ± 0.003	0.0025 ± 0.0149	0.146 ± 0.015	174 ± 5
4 tags	30	27	90.0%	[74.4, 96.5]	0.138 ± 0.004	0.0006 ± 0.0202	0.125 ± 0.020	211 ± 38

5 tags	30	17	56.7%	[39.2, 72.6]	0.125 ± 0.003	-0.0010 ± 0.0186	0.102 ± 0.020	276 ± 61
6 tags	30	5	16.7%	[7.3, 33.6]	0.114 ± 0.004	-0.0075 ± 0.0204	0.083 ± 0.018	350 ± 46
8 tags	30	2	6.7%	[1.8, 21.3]	0.098 ± 0.003	0.0014 ± 0.0205	0.064 ± 0.021	412 ± 32

Table S10. *Multi-tag study S2: per-condition raw data summary (n = 210; success rate vs. inter-tag separation Δ at fixed N = 3, coaxial alignment, air; 30 repetitions per condition).*

Condition	n	k	Success	95% Wilson CI	Signal (mean ± SD)	Noise (mean ± SD)	Score (mean ± SD)	Time, ms (mean ± SD)
5 mm	30	0	0.0%	[0.0, 11.4]	0.095 ± 0.003	0.0016 ± 0.0114	0.055 ± 0.012	527 ± 5
10 mm	30	6	20.0%	[9.5, 37.3]	0.110 ± 0.004	0.0042 ± 0.0098	0.088 ± 0.013	480 ± 41
15 mm	30	26	86.7%	[70.3, 94.7]	0.126 ± 0.004	0.0025 ± 0.0110	0.113 ± 0.012	387 ± 34
20 mm	30	30	100.0%	[88.6, 100.0]	0.140 ± 0.003	-0.0003 ± 0.0103	0.131 ± 0.012	350 ± 6
25 mm	30	30	100.0%	[88.6, 100.0]	0.156 ± 0.004	-0.0004 ± 0.0080	0.149 ± 0.010	326 ± 6
30 mm	30	30	100.0%	[88.6, 100.0]	0.171 ± 0.004	0.0015 ± 0.0074	0.170 ± 0.010	299 ± 8
40 mm	30	30	100.0%	[88.6, 100.0]	0.199 ± 0.004	0.0013 ± 0.0068	0.201 ± 0.009	249 ± 7

Table S11. *Multi-tag study S3: per-condition raw data summary (n = 120; success rate vs. transponder angle θ_t at fixed N = 3, Δ = 20 mm, in 0.9% NaCl; 30 repetitions per condition).*

Condition	n	k	Success	95% Wilson CI	Signal (mean ± SD)	Noise (mean ± SD)	Score (mean ± SD)	Time, ms (mean ± SD)
0°	30	30	100.0%	[88.6, 100.0]	0.170 ± 0.003	-0.0019 ± 0.0076	0.169 ± 0.009	181 ± 5
30°	30	17	56.7%	[39.2, 72.6]	0.147 ± 0.003	-0.0011 ± 0.0123	0.101 ± 0.013	242 ± 45
60°	30	0	0.0%	[0.0, 11.4]	0.085 ± 0.003	-0.0010 ± 0.0158	-0.005 ± 0.015	317 ± 6
90°	30	0	0.0%	[0.0, 11.4]	-0.001 ± 0.003	0.0014 ± 0.0115	-0.134 ± 0.013	341 ± 4

S9. Statistical Analysis Scripts

This section provides the statistical analysis scripts that reproduce the analyses reported in the main manuscript. Listing S1 (multi-tag) reproduces the per-condition success rate aggregation, Wilson 95% confidence intervals, logistic regression operating thresholds (N_{50}, Δ_{50}), and binomial-MLE angular fit for the multi-tag dataset reported in §3.7 and Tables S9–S11. Listing S2 (Kalman) reproduces the per-trajectory RMSE/bias/HF noise reduction metrics of §3.7, the Q_vel sensitivity sweep of Table S13, and the v2 re-tuning analysis. The complete reproduction package—including the raw datasets (intellisense_dataset_primary.xlsx, 480 rows × {session, condition, medium, angle_deg, encapsulation, repetition, distance_mm, rssi_db}; intellisense_dataset_multitag.xlsx, 540 rows; kalman_validation_dataset.xlsx, 1950 rows), both Python scripts, and the analogous R script for the primary 480-measurement dataset, the bootstrap-confidence-interval, mixed-effects ANOVA, non-linear least-squares angular fit, path-loss regression, GUM uncertainty propagation, and Bland–Altman agreement analyses reported in §3.6 of the main manuscript—are available from the corresponding author upon reasonable request, in conformity with the Data Availability Statement of the main manuscript.

Listing S1. *Multi-tag analysis script (Python). The script aggregates per-condition success rates with Wilson 95% confidence intervals, fits the logistic operating-threshold models, and recovers the angular operating envelope by binomial MLE. Inputs: the raw dataset of 540 multi-tag measurements (intellisense_dataset_multitag.xlsx). Outputs:*

per-study summary tables (reproducing the multi-tag results of §3.7), the operating thresholds N_{50}, Δ_{50}, and the multi-tag angular θ_{50}.

```
# Listing S1 - Multi-tag analysis script (Python 3.12+)
# Reproduces §3.7 multi-tag results and operating-envelope thresholds
# of Section 3.7 of the main manuscript from the raw dataset
# 'intellisense_dataset_multitag.xlsx' (n = 540 measurements).
#
# Required packages: pandas, numpy, scipy, matplotlib

import pandas as pd
import numpy as np
import matplotlib.pyplot as plt
from scipy.optimize import curve_fit, minimize
from scipy import stats

df = pd.read_excel('intellisense_dataset_multitag.xlsx')

def wilson_ci(k, n, alpha=0.05):
    z = stats.norm.ppf(1 - alpha/2)
    p = k/n
    denom = 1 + z**2/n
    centre = (p + z**2/(2*n)) / denom
    halfw = z * np.sqrt(p*(1-p)/n + z**2/(4*n**2)) / denom
    return (max(0, centre - halfw), min(1, centre + halfw))

def logistic(x, x0, k):
    return 1.0 / (1.0 + np.exp(-k * (x - x0)))

# Per-condition success-rate aggregation (S1, S2, S3) for §3.7 of the main manuscript
for study in ['S1', 'S2', 'S3']:
    sub = df[df['study'] == study]
    summary = sub.groupby('param').agg(
        k=('success', 'sum'), n=('rep', 'count'),
        time_mean=('time_ms', 'mean'), time_sd=('time_ms', 'std')
    ).reset_index()
    print(f'\n=== Study {study} ===')
    for _, r in summary.iterrows():
        lo, hi = wilson_ci(int(r['k']), int(r['n']))
        print(f"{r['param']:>5} | {r['k']}/{r['n']} | "
              f"{100*r['k']/r['n']:.1f}% [{100*lo:.1f}, {100*hi:.1f}] | "
              f"time = {r['time_mean']:.0f} +/- {r['time_sd']:.0f} ms")

# Logistic regression (operating thresholds N50, Delta50)
for study, monotone in [('S1', -1), ('S2', +1)]:
    s = df[df['study'] == study].groupby('param').agg(
        k=('success', 'sum'), n=('rep', 'count')).reset_index()
    x = monotone * s['param'].values.astype(float)
    y = (s['k'] / s['n']).values
    popt, _ = curve_fit(logistic, x, y, p0=[0, 1], maxfev=5000)
    x50 = monotone * popt[0]
    x95 = monotone * (popt[0] + np.log(0.95/0.05) / popt[1])
    print(f'{study}: x50 = {x50:.2f}, x95 = {x95:.2f}')

# S3 angular fit via binomial MLE (logistic likelihood)
def neg_log_lik(params, x, k, n):
    p = np.clip(logistic(x, *params), 1e-10, 1 - 1e-10)
    return -np.sum(k * np.log(p) + (n - k) * np.log(1 - p))

s3 = df[df['study'] == 'S3'].groupby('param').agg(
    k=('success', 'sum'), n=('rep', 'count')).reset_index()
x_s3 = -s3['param'].values.astype(float)
result = minimize(neg_log_lik, [-30, 0.15],
                  args=(x_s3, s3['k'].values.astype(float),
                        s3['n'].values.astype(float)),
                  method='Nelder-Mead')
x0_fit, slope_fit = result.x
print(f'S3 angle50 = {-x0_fit:.1f} deg, slope = {slope_fit:.3f}')
```

```
# Multi-tag figure (3 panels) reproduction is provided in the article supplement
# repository alongside the raw dataset. Identical plot code yields
# the multi-tag operating-envelope panels of §3.7.
```

Listing S2. *Kalman validation analysis script (Python). Reproduces the per-trajectory RMSE/bias/HF noise reduction metrics of §3.7 (main manuscript) and the Q_vel sensitivity sweep of Table S13 (this supplement) from the raw dataset of 1950 Kalman validation samples (kalman_validation_dataset.xlsx). The constant-velocity Kalman filter implementation is shown explicitly for reproducibility; the v2 re-tuned configuration uses Q_vel = 50 mm²/s², a ~150× increase relative to the firmware as-shipped value (Q_vel ≈ 0.3 mm²/s²).*

```
# Listing S2 - Kalman validation analysis script (Python 3.12+)
# Reproduces §3.7 Kalman results, Tables S12-S13, and the Q-sweep
# of Section 3.7 (main manuscript) and Section S10 (supplement)
# from the raw dataset 'kalman_validation_dataset.xlsx' (n = 1950).
#
# Required packages: pandas, numpy, matplotlib

import pandas as pd
import numpy as np

df = pd.read_excel('kalman_validation_dataset.xlsx')
dt = 0.02  # 50 Hz sampling
sigma_raw = 3.017  # RSSI inversion noise (mm), calibrated empirically

def run_kalman_cv(r_raw, dt, Q_vel, R_meas,
                  Q_pos=0.01, P0_pos=10, P0_vel=100):
    """Constant-velocity Kalman filter on distance measurements."""
    n = len(r_raw)
    F = np.array([[1, dt], [0, 1]])
    H = np.array([[1, 0]])
    Q = np.array([[Q_pos, 0], [0, Q_vel]])
    R = np.array([[R_meas]])
    x = np.array([r_raw[0], 0.0])
    P = np.array([[P0_pos, 0], [0, P0_vel]])
    estimates = np.zeros(n)
    estimates[0] = x[0]
    for k in range(1, n):
        x = F @ x; P = F @ P @ F.T + Q
        z = r_raw[k]
        y_innov = z - H @ x
        S = H @ P @ H.T + R
        K = P @ H.T @ np.linalg.inv(S)
        x = x + (K @ y_innov.reshape(1)).flatten()
        P = (np.eye(2) - K @ H) @ P
        estimates[k] = x[0]
    return estimates

# Wrap-exclusion regions (post-discontinuity transients)
wrap_info = {
    'T1': {'wraps': [0, 50, 100, 150, 200], 'excl': 10},
    'T2': {'wraps': [0, 25, 50, 75], 'excl': 5},
    'T3': {'wraps': [0], 'excl': 30},
    'T4': {'wraps': [50], 'excl': 5},
}

Q_VEL_V2 = 50  # firmware v2 tuning

for traj_id in ['T1', 'T2', 'T3', 'T4']:
    sub = df[df['trajectory'] == traj_id]
    r_raw = sub['r_raw_mm'].values
    r_true = sub['r_true_mm'].values
    r_kal_v1 = sub['r_kalman_mm'].values  # firmware as-shipped
    info = wrap_info[traj_id]

    # Build steady-state mask
    n = len(r_raw)
    mask = np.ones(n, dtype=bool)
```

```
    for w in info['wraps']:
        mask[w:w + info['excl']] = False

    # Run re-tuned v2 filter
    r_kal_v2 = run_kalman_cv(r_raw, dt, Q_VEL_V2, sigma_raw**2)

    # Compute steady-state metrics
    print(f'\n=== {traj_id} ===')
    for name, est in [('raw', r_raw),
                      ('moving_avg', sub['r_moving_avg_mm'].values),
                      ('kalman_v1', r_kal_v1),
                      ('kalman_v2', r_kal_v2)]:
        err = est[mask] - r_true[mask]
        rmse = np.sqrt(np.mean(err**2))
        bias = err.mean()
        # HF noise reduction (diff-based, full trajectory)
        if name != 'raw':
            diff_raw = np.diff(r_raw - r_true)
            diff_est = np.diff(est - r_true)
            hf_red = (1 - np.var(diff_est)/np.var(diff_raw)) * 100
            print(f'  {name:12s}: RMSE={rmse:.3f} mm, bias={bias:+.3f},
HF_red={hf_red:+.1f}%')
        else:
            print(f'  {name:12s}: RMSE={rmse:.3f} mm, bias={bias:+.3f}')

# Q_vel sensitivity sweep on T1 (Table S13)
sub_t1 = df[df['trajectory'] == 'T1']
r_raw_t1 = sub_t1['r_raw_mm'].values
r_true_t1 = sub_t1['r_true_mm'].values
mask_t1 = np.ones(250, dtype=bool)
for w in [0, 50, 100, 150, 200]:
    mask_t1[w:w+10] = False

print('\n=== Q_vel sweep on T1 ===')
for Q_vel in [0.1, 0.3, 1, 3, 10, 30, 50, 100, 200]:
    est = run_kalman_cv(r_raw_t1, dt, Q_vel, sigma_raw**2)
    err = est[mask_t1] - r_true_t1[mask_t1]
    rmse = np.sqrt(np.mean(err**2))
    bias = err.mean()
    lag_ms = -bias / 20 * 1000  # at v=20 mm/s
    print(f'  Q_vel={Q_vel:6.1f}: RMSE={rmse:.3f}, lag={lag_ms:+.0f} ms')
```

S10. Kalman Filter Validation Dataset (Secondary/Offline Analysis)

This supplement provides the raw data summary and the Q-tuning sensitivity analysis underlying the Kalman filter validation reported in §3.7 of the main manuscript. Two complementary tables are presented: Table S12 documents the four pre-defined trajectory datasets that constitute the validation corpus (n = 1950 samples total at 50 Hz sampling), and Table S13 provides the Q_vel sensitivity sweep on the T1 trajectory that motivated the firmware v2 re-tuning recommendation. The complete sample-by-sample dataset (1950 rows × 8 columns: trajectory, sample, time_s, r_true_mm, rssi_db, r_raw_mm, r_moving_avg_mm, r_kalman_mm) is provided as the auxiliary file kalman_validation_dataset.xlsx alongside the main supplementary material.

Table S12 documents the four predefined trajectories used for secondary estimator analysis. The dataset was acquired/resampled at 50 Hz for the offline comparison of raw RSSI-derived distance, moving average, and Kalman configurations. This 50 Hz analysis rate is distinct from

the measured end-to-end NFC detection response of 45 ± 8 ms (approximately 22 Hz) reported in the main manuscript.

Table S12. *Kalman validation dataset: trajectory characteristics. Four pre-defined trajectories spanning the dynamic motion regimes relevant to laparoscopic surgical scanning. Distance range, RSSI statistics, and the post-discontinuity transient regions excluded from steady-state metric computation are reported per trajectory.*

Trajectory	Description	n samples	Duration	Distance range	RSSI mean ± SD (dB)	Excluded samples
T1	Constant velocity 20 mm/s sawtooth (5 cycles)	250	5.0 s	10–30 mm	-78.3 ± 4.7	50 (5 × 10)
T2	Constant velocity 50 mm/s sawtooth (4 cycles)	100	2.0 s	10–29 mm	-78.1 ± 4.7	20 (4 × 5)
T3	Sinusoidal 0.5 Hz, ±10 mm	1500	30.0 s	5–25 mm	-73.4 ± 7.5	30 (1 × 30)
T4	Step response 25→10 mm at t=1 s	100	2.0 s	10–25 mm	-75.9 ± 6.3	5 (1 × 5)

Table S13 reports the Q_vel sensitivity sweep on T1. The sweep is an offline estimator-development exercise and is not treated as validation of real-time 3D localisation. Its purpose is to quantify the noise–lag trade-off for future firmware development.

Three observations support the firmware v2 tuning recommendation. First, the lag transitions sharply between Q_vel = 10 mm²/s² (37 ms) and Q_vel = 50 mm²/s² (3 ms), with the lag-versus-Q_vel relationship following the classical first-order Kalman time-constant scaling $\tau \propto$ (R/Q_vel)^½. Second, the HF noise reduction (computed as the variance reduction of the differentiated error sequence relative to the raw RSSI baseline) remains essentially constant across the entire Q_vel range (70.6–74.7%), confirming that the noise-rejection capability is limited by the measurement noise covariance R rather than by the process noise covariance Q. Third, RMSE decreases monotonically as Q_vel increases up to Q_vel ≈ 100–200 mm²/s², suggesting the optimum tuning for this dataset lies near the upper bound of the sweep range; the conservative selection of Q_vel = 50 mm²/s² for the firmware v2 release reflects an additional safety margin against potential overshoot on rapid step transitions (T4) and the inter-batch variability of the path-loss calibration.

Table S13. *Q_vel sensitivity sweep on T1 (constant-velocity 20 mm/s sawtooth, 200 samples after wrap exclusion)—idealised offline simulation via run_kalman_cv() (Listing S2). RMSE, bias, residual standard deviation, high-frequency noise reduction (variance ratio of differentiated error to raw-RSSI baseline), and steady-state tracking lag (computed from bias/probe velocity). Bold rows mark the design-intent values: Q_vel ≈ 0.3 (firmware v1 design intent) and Q_vel = 50 (firmware v2 re-tuning). The actual firmware-as-shipped output stored in the validation dataset (column r_kalman_mm) deviates from the idealised Q_vel = 0.3 simulation row of this table due to firmware implementation factors (fixed-point arithmetic, sample timing quantisation, internal state initialisation) not captured by the offline Python simulation. Full per-trajectory firmware-output metrics across T1–T4 are tabulated in Table S12bis below.*

Q_vel (mm²/s²)	RMSE (mm)	Bias (mm)	Std (mm)	HF noise reduction	Lag (ms)
0.1	3.048	-1.412	2.701	+70.6%	+71
**0.3 (firmware v1)**	**2.790**	**-1.556**	**2.315**	**+70.9%**	**+78**
1.0	2.513	-1.558	1.972	+71.5%	+78
3.0	2.403	-1.287	2.029	+72.3%	+64
10.0	2.318	-0.747	2.194	+73.3%	+37
30.0	2.070	-0.252	2.055	+74.1%	+13
**50.0 (re-tuned v2)**	**1.926**	**-0.056**	**1.926**	**+74.4%**	**+3**
100.0	1.758	+0.168	1.750	+74.7%	-8

200.0	1.673	+0.335	1.639	+74.7%	-17

Table S12bis reports secondary per-trajectory metrics for the four estimator variants. The Kalman v2 results are generated by offline re-simulation with Q_vel = 50 mm^2/s^2. These results support firmware tuning only; they are not used as primary sensing-performance endpoints.

Table S12bis. *Per-trajectory firmware-output metrics for the four validation trajectories and four estimator variants. RMSE, bias, lag, and HF noise reduction computed on the steady-state mask of Table S12. Kalman v1: firmware-output column r_kalman_mm. Kalman v2: offline re-simulation with Q_vel = 50 mm^2/s^2 (Listing S2). HF noise reduction = variance ratio of differentiated error to raw-RSSI baseline. Lag = −bias / probe velocity, reported only where the constant-velocity concept applies.*

Trajectory	Filter	RMSE (mm)	Bias (mm)	Lag (ms)	HF noise reduction
T1 (20 mm/s sawtooth)	Raw RSSI	3.293	+0.520	n/a	baseline (0%)
T1	Moving avg (8 ms)	1.239	−0.369	+18	+70.9%
**T1**	**Kalman v1 (firmware)**	**3.302**	**−2.966**	**+148**	**+70.7%**
**T1**	**Kalman v2 (Q=50)**	**1.926**	**−0.059**	**+3**	**+74.4%**
T2 (50 mm/s sawtooth)	Raw RSSI	3.227	+0.438	n/a	baseline (0%)
T2	Moving avg (8 ms)	4.445	+0.301	−6	+41.8%
**T2**	**Kalman v1 (firmware)**	**5.535**	**−1.369**	**+27**	**+32.8%**
**T2**	**Kalman v2 (Q=50)**	**4.846**	**+0.566**	**−11**	**+44.0%**
T3 (sinusoidal 0.5 Hz)	Raw RSSI	2.584	+0.092	n/a	baseline (0%)
T3	Moving avg (8 ms)	1.447	+0.107	n/a	+95.9%
**T3**	**Kalman v1 (firmware)**	**3.745**	**+0.245**	**n/a**	**+99.1%**
**T3**	**Kalman v2 (Q=50)**	**1.325**	**+0.088**	**n/a**	**+95.9%**
T4 (step 25→10 mm)	Raw RSSI	2.805	+0.086	n/a	baseline (0%)
T4	Moving avg (8 ms)	0.946	+0.125	n/a	+86.3%
**T4**	**Kalman v1 (firmware)**	**2.344**	**−1.117**	**n/a**	**+88.7%**
**T4**	**Kalman v2 (Q=50)**	**1.278**	**−0.200**	**n/a**	**+83.6%**

S11. AC Resistance and Idealised Unloaded-Q Estimate

This section retains the closed-form conductor-loss calculation as an engineering design estimate. The resulting unloaded-Q value is an idealised upper bound because the model does not fully include proximity effect, winding self-capacitance, solder/interconnect loss, matching-network loss, reader loading, or the complete frequency-dependent complex permeability of the fabricated ferrite core. The measured operational loaded Q = 23 reported in the main manuscript is the experimentally relevant quantity.

S11.1 DC and Skin-Depth Analysis

The DC resistance of the winding is determined from the conductor geometry. The total wire length comprises 20 turns at a mean coil circumference of π × (D + d_wire) = π × 3.2 × 10^{-3} m,

yielding a total wire length l_{wire} = 0.20 m. With a 0.2 mm diameter copper wire (cross-sectional area $A_{wire} = \pi \times (10^{-4})^2 = 3.14 \times 10^{-8}$ m²) and bulk copper resistivity $\rho_{Cu} = 1.72 \times 10^{-8}$ Ω·m at 20 °C, the DC resistance is

$$R_{DC} = \rho_{Cu} \cdot l_{wire} / A_{wire} = (1.72 \times 10^{-8} \times 0.20) / (3.14 \times 10^{-8}) \approx 110\ \text{m}\Omega \quad \text{(S11.1)}$$

At 13.56 MHz, the electromagnetic skin depth in copper is

$$\delta_{Cu} = \sqrt{(2 \rho_{Cu} / \omega_0 \mu_0)} = \sqrt{[(2 \times 1.72 \times 10^{-8}) / (2\pi \times 13.56 \times 10^6 \times 4\pi \times 10^{-7})]} \approx 17.9\ \mu\text{m} \quad \text{(S11.2)}$$

For the 0.2 mm diameter wire, the conductor radius-to-skin-depth ratio r_w/δ_{Cu} = 100/17.9 ≈ 5.6 places the wire firmly in the high-frequency regime where the skin effect dominates the current distribution. The classical Howe formula for round-conductor AC-to-DC resistance ratio applies,

$$R_{AC} / R_{DC} \approx (r_w / 2\delta_{Cu}) + 0.25 + 3\ \delta_{Cu} / (8\ r_w) \approx 2.80 + 0.25 + 0.067 \approx 3.12 \quad \text{(S11.3)}$$

yielding R_{AC} ≈ 343 mΩ under the selected closed-form approximation. The proximity effect for the tight single-layer winding is not independently measured; therefore, the calculated R_{AC} should be treated as an order-of-magnitude design estimate rather than a complete RF loss budget.

S11.2 Ferrite Magnetic Losses and Quality Factor

A ferrite-loss term was included in the original analytical model using an assumed magnetic-loss tangent at 13.56 MHz. Because the exact frequency-dependent complex permeability of the individual fabricated rod was not independently measured and the manufacturer information used here does not establish the complete antenna loss budget, the resulting ferrite-loss resistance is retained only as a sensitivity/design estimate.

$$R_{ferrite,model} = \omega_0 \cdot L_{ant} \cdot \tan\delta_\mu \cdot (\mu_{eff} / \mu_i) \quad \text{(S11.4)}$$

Combining the simplified conductor and ferrite-loss terms gives an idealised unloaded-Q estimate of order 10^3 (approximately 3700 with the original input assumptions). This value is not claimed as a measured antenna property and is not used to assert a 37–74-fold hardware performance improvement over the preceding reference antenna. The operational matched-network measurement, Q_{loaded} = 23, is the appropriate experimentally supported Q value for the present prototype.

S11.3 Local Parametric Sensitivity of the Closed-Form Antenna Model

To quantify the local robustness of the closed-form design equations without assigning unsupported manufacturing tolerances, each represented input was perturbed independently by ±5% around the nominal design point (Lrod = 25 mm, D = 3 mm, μi = 40, N = 20, winding length lc = 4 mm, I = 100 mA). Relative to the nominal μeff = 19.58, Lant = 17.39 μH, and H (16.45 mm) = 5.00 A/m, a ±5% perturbation of μi changes μeff, Lant, and H by approximately −2.45/+2.32%; a ±5% change in rod length changes μeff and Lant by −3.90/+3.74% and H at the fixed 16.45 mm centre-referenced distance by +1.48/−1.83%; a ±5% change in rod diameter changes μeff by +3.94/−3.71% and changes Lant and H by approximately −6.20/+6.16%; and a ±5% change in winding length changes Lant by +5.26/−4.76% while leaving the magnetic-moment field expression unchanged. A ±5% excitation current perturbation changes H linearly by ±5%. These values are local model sensitivities, not statistical uncertainty intervals. The closed-form formulation assumes a centred winding and frequency-independent effective material parameters at the design point; winding offset sensitivity and frequency dispersion therefore require dedicated FEM/material-characterisation sweeps and are not claimed to be

quantified here. The measured Lant deviation (−2.9%) and 4.2% RMS analytical–FEM field-profile difference provide aggregate experimental/numerical checks on the scale of the remaining model mismatch.

S12. Magnetic Moment and Axial-Field Profile—Full Derivation

This section presents the analytical magnetic moment and finite rod field model together with the empirical transverse-coupling term. The axial field profile is physics-derived from stated inputs; the field-to-detection mapping is hybrid because the excitation current is a modelling input and $\eta_\perp$ is empirically anchored.

S12.1 Equivalent Magnetic Moment

The equivalent magnetic moment provides a convenient analytical representation of the field profile. It should be interpreted as a model quantity based on the assumed nominal antenna current rather than as a directly calibrated magnetic-dipole moment of the fabricated device.

$$m = N \cdot I \cdot \mu_{eff} \cdot A_{core} \quad \text{(S12.1)}$$

Using the nominal modelling input I_{reader} = 100 mA gives

$$m = 20 \times 0.100 \times 19.58 \times (7.07 \times 10^{-6}) \approx 0.277 \times 10^{-3}\ \text{A·m}^2 \quad \text{(S12.2)}$$

S12.2 Axial Field Profile

The axial magnetic field intensity along the antenna axis is approximated by the finite rod dipole formula:

$$H(r) \approx m / [2\pi \cdot (r^2 + (L/2)^2)^{(3/2)}] \quad \text{(S12.3)}$$

The finite rod correction is important because the experimental range is comparable with L/2. The expression is used to capture the field profile shape; absolute field-to-detection prediction additionally depends on excitation calibration and the empirically anchored system threshold.

S12.3 Angular Coupling Formulation

The angular dependence of the coupling at non-coaxial transponder orientations is governed by the projection of the antenna magnetic field onto the transponder coil normal. For a transponder oriented at angle θ to the antenna axis, the effective coupled field is

$$H_{coupled}(r, \theta) = H(r) \cdot \cos(\theta) + H_\perp(r) \cdot \sin(\theta) \cdot \eta_\perp \quad \text{(S12.4)}$$

Here $\eta_\perp$ is an empirical coupling coefficient introduced to represent the non-zero orthogonal response. Because $\eta_\perp$ is obtained from experimental anchoring, recovery of the 90° point is not an independent model validation. A held-out prediction requires parameter calibration on a predefined subset and testing on measurements excluded from that calibration.

S13. Matching Network and Reader Coupling—Full Derivation

This section describes the reader-side resonance/matching model. The analysis distinguishes the 50 Ω reference used by VNA instrumentation from the actual differential output behaviour of the ST25R3916B antenna driver.

S13.1 L-Network Topology

The ferrite coil impedance is highly reactive at 13.56 MHz and requires a series/parallel matching network at the ST25R3916B antenna terminals. The reader IC is not modelled as a fixed 50 Ω differential source. Instead, component values are selected using the reader output model, antenna impedance, and measured VNA response. The 50 Ω quantity applies only to the VNA measurement reference plane.

Initial analytical targets: $C_s \approx 7.9$ pF and $C_p \approx 90$ pF; final operational values are verified by the measured matched network resonance and Q. (S13.1)

S13.2 Harmonic Balance Simulation

Circuit simulation was used to choose initial resonance/matching values and to explore loading/Q sensitivity. The simulation should be interpreted as a reader network design model rather than as a 50 Ω source transformation. Final evidence is provided by the as-built VNA measurements at the 50 Ω instrumentation reference plane (main manuscript Table 3).

S13.3 Q_loaded Trade-off Analysis

The practical loaded quality factor is determined by the complete antenna, matching network, reader loading, and signalling requirements. Operational performance is therefore represented by the measured $Q_{loaded} = 23$. Component tolerance simulations are retained as design-planning information only, and do not replace hardware measurements.

S14. Methodological Readiness Model-Based Projections

This section contains model-based planning exercises only. The values are generated from simulated or model-derived data and are not measurements of the present single-axis prototype. They are retained to document a prospective methodology for future multi-axis/ex vivo work, and must not be interpreted as validation of 3D localisation performance.

S14.1 Localisation Error Parameter Recovery

The reported parameter-recovery exercise uses simulated 3D observations generated from the assumed localisation error model. Agreement between recovered and generating parameters is therefore an implementation consistency check, not independent experimental validation. These quantities are not used in the main manuscript to substantiate prototype localisation accuracy.

S14.2 Three-Dimensional Bland–Altman Agreement

The simulated Bland–Altman exercise gives X bias +0.025 mm with 95% LoA [−2.31, +2.36] mm, Y bias −0.013 mm with 95% LoA [−2.34, +2.32] mm, and Z bias −0.59 mm with 95% LoA [−4.22, +3.05] mm. These limits are substantially wider than ±0.5 mm. Because the dataset is simulated, these LoA are planning outputs only, and are not interpreted as experimental localisation accuracy.

S14.3 Prospective Sample Size Calculation

The prospective sample size calculations are illustrative planning estimates based on effect sizes observed within one prototype, and therefore may be optimistic for a multi-prototype, multi-operator ex vivo study. The values should be recalculated once pilot ex vivo variance components are available; the present estimates are not used to claim that a future study is already adequately powered.

S15. Multi-Layer Phantom Fabrication Protocols and Quality Control

This supplementary section provides the complete fabrication protocols and quality control records for the three independent multi-layer phantom batches (B1, B2, B3) used in Section 3.8's detection performance characterisation. The protocols achieve quantitative reproducibility against IT'IS Foundation database 4.1 reference values at 13.56 MHz (deviations +0.7% to +1.4% in permittivity, +0.7% to +1.3% in conductivity; n = 180 measurements per stratum).

S15.1 Phantom Materials and Composition

Five tissue-equivalent strata were formulated to replicate the colorectal wall layered structure. Aqueous strata (mucosa, submucosa, muscularis propria, serosa) comprised agar-based gels with NaCl content adjusted per stratum to achieve the target conductivity. The submucosa stratum incorporated graphite powder (5.0% w/v, granulometry < 50 μm, AlfaAesar 13881) to elevate permittivity to the target $\varepsilon_r = 65 \pm 5$. The perirectal fat stratum employed an oil-in-gelatin emulsion stabilised with surfactant. All reagents were food grade or analytical grade, as specified in Table S14.

Table S14. *Phantom material composition specifications per stratum.*

Stratum	Base material	NaCl content	Special additives	Target ε_r	Target σ [S/m]
Mucosa	Agar 2.0% w/v	1.2% w/v	—	85	1.0
Submucosa	Agar 2.0% w/v	1.0% w/v	Graphite 5.0% w/v	65	0.8
Muscularis propria	Agar 2.5% w/v	0.8% w/v	—	58	0.6
Serosa	Agar 2.5% w/v	0.6% w/v	—	52	0.5
Perirectal fat	Gelatin 15% / Sunflower oil 70%	—	Tween 80 (2% v/v)	5.5	0.04

S15.2 Fabrication Procedure

All aqueous strata were prepared by dissolving agar in deionised water at 90°C with continuous stirring for 15 minutes, followed by NaCl addition during the cooling phase (60°C). The graphite powder for the submucosa stratum was incorporated at 60°C with high-shear mixing (5 minutes at 5,000 RPM) to ensure homogeneous dispersion before pouring into the layer mould (95 × 60 × variable mm acrylic frame with adjustable depth). Each layer was poured sequentially upon partial gelling of the underlying layer (8–10 minutes at 22 °C ambient) to ensure mechanical adhesion without inter-layer mixing.

The perirectal fat emulsion was prepared in a separate workflow: the gelatin was dissolved in 30% v/v aqueous phase at 50°C, then combined with the 70% v/v sunflower oil and Tween 80 surfactant. The mixture was emulsified using a high-shear blender (Silverson L5M) at 10,000 RPM for 5 minutes to achieve a stable oil-in-gelatin emulsion with droplet size < 100 μm (verified by optical microscopy, n = 100 droplets per batch). The emulsion was poured into the bottom of the phantom mould before the aqueous strata to allow complete gelling at 4 °C for 30 minutes prior to the aqueous strata sequence.

S15.3 Per-Batch Quality Control Record

Each of the three independent batches (B1, B2, B3) underwent a standardised quality control protocol immediately upon fabrication completion: (i) visual inspection for layer uniformity, air bubble inclusions, and inter-layer mixing; (ii) thickness verification at five positions per layer

using digital callipers (accuracy ±0.05 mm); (iii) dielectric verification at four post-fabrication timepoints (30 min, 1 h, 2 h, 4 h) via open-ended coaxial probe; (iv) temperature stability monitoring during the 4-hour operating window. Table S15 summarises the QC metrics across the three batches.

Table S15. *Per-batch quality control record for the three independent multi-layer phantoms.*

QC parameter	Specification	B1	B2	B3	Status
Total fabrication time	≤ 6 h	5.5 h	5.7 h	5.8 h	✓ All pass
Layer thickness deviation	≤ ±0.2 mm	±0.15 mm	±0.18 mm	±0.16 mm	✓ All pass
Air-bubble inclusions	None visible (>0.5 mm)	0	0	1 (<1 mm)*	✓ All pass
Inter-layer adhesion	No separation	Pass	Pass	Pass	✓ All pass
Dielectric drift @ 4h	< 5%	1.4%	1.7%	1.8%	✓ All pass
Temperature stability	±1°C	±0.4°C	±0.5°C	±0.4°C	✓ All pass
Inter-batch CV (ε_r)	< 5%	—	—	—	0.91% ✓
Inter-batch CV (σ)	< 5%	—	—	—	1.43% ✓

**Single sub-millimetre bubble in B3 perirectal fat stratum, located outside the active detection zone (verified prior to detection measurements). All other QC parameters within specification across all three batches.*

S16. Multi-Layer Phantom Detection—Per-Cell Results and Exploratory Statistical Analyses

This supplementary section provides the complete per-cell detection statistics, ANOVA decomposition, and multiple comparisons-corrected pairwise tests underlying the Section 3.8 detection performance characterisation. Total measurements: n = 1440 at 22°C (3 batches × 30 reps × 16 cells) plus n = 600 at 37°C (3 batches × 30 reps × 2 coaxial cells + 1 batch × 30 reps × 14 non-coaxial cells = 180 + 420 = 600).

S16.1 Per-Cell Detection Probability and Conditional Detection Distance

Table S16 reports two distinct outcomes: detection success (n_succ/n_total) for every configuration and detection distance only among successful trials. This separation is essential because zero-detection cells do not have a continuously observed r_det endpoint. The 22 °C dataset includes three batches, whereas the 37 °C non-coaxial subset includes batch B1 only; cross-temperature comparisons in those cells are therefore not batch-independent.

Table S16. *Per-cell detection statistics at 22 °C and 37 °C across the multi-layer phantom configuration matrix.*

fat [mm]	θ_t	T [°C]	n_succ/n_tot	Mean [mm]	SD [mm]	95% BCa CI	Shapiro–Wilk p
0	0°	22	90/90	16.228	0.276	[16.17, 16.28]	0.528
0	0°	37	90/90	16.168	0.306	[16.10, 16.23]	0.773
0	30°	22	90/90	15.155	0.392	[15.08, 15.23]	0.858
0	30°	37	30/30	15.237	0.378	[15.11, 15.37]	0.874
0	60°	22	90/90	11.895	0.509	[11.79, 12.00]	0.384
0	60°	37	30/30	11.789	0.532	[11.61, 11.98]	1.000
0	90°	22	78/90	6.028	0.397	[5.94, 6.12]	0.299
0	90°	37	27/30	6.004	0.475	[5.84, 6.19]	0.531
10	0°	22	90/90	16.158	0.301	[16.10, 16.22]	0.727
10	0°	37	89/90	16.065	0.322	[16.00, 16.13]	0.842
10	30°	22	17/90	15.514	0.157	[15.44, 15.59]	0.955
10	30°	37	7/30	15.536	0.273	[15.40, 15.80]	0.136
10	60°	22	0/90	—	—	Wilson UB:	—

						4.09%	
10	60°	37	0/30	—	—	Wilson UB: 11.35%	—
10	90°	22+37	0/120	—	—	Wilson UB: 3.10%	—
20	all	22+37	0/480	—	—	Wilson UB: 0.78%	—
30	all	22+37	0/480	—	—	Wilson UB: 0.78%	—

All Shapiro–Wilk p-values exceed 0.05, confirming approximate normality of detection range distributions in detectable cells, supporting the validity of parametric ANOVA and Welch t-test inference. Wilson upper bounds for non-detectable cells provide statistically robust limits on the residual probability of successful detection.

S16.2 Exploratory Factorial Analysis of Detectable Observations

The three-way ANOVA is reported only as an exploratory summary of available detection distance observations. It is not considered the primary inferential model because the design is unbalanced across temperature/batch and several configurations have zero or partial detection. Primary interpretation is therefore based on the binomial success proportions and conditional range summaries in Table S16. A future raw data reanalysis could use a binomial mixed-effects model for detection probability and a separate mixed model for r_{det} conditional on success, with prototype/tag/batch/session effects included when identifiable.

Table S17. Exploratory ANOVA on available detectable observations. Because the design is unbalanced and detection is incomplete in some cells, this table is descriptive and is not used for the primary inference.

Source	SS	df	F	p	η^2_p	Cohen's f
Thickness	3.218	1	26.47	<0.001	0.031	0.179
Angle	5492.135	2	22,589.99	<0.001	0.982	7.387
Temperature	0.174	1	1.43	0.231	0.002	0.042
Thickness × Angle	0.036	2	0.15	0.864	0.0004	0.019
Thickness × Temperature	0.126	1	1.03	0.309	0.001	0.035
Angle × Temperature	0.201	2	0.83	0.438	0.002	0.045
Three-way interaction	0.116	2	0.48	0.622	0.001	0.034
Residual	100.653	828	—	—	—	—

The angular factor dominates the variance decomposition ($\eta^2_p = 0.982$, Cohen's f = 7.387, an extremely large effect by Cohen's conventions of f = 0.10/0.25/0.40 for small/medium/large effects). The thickness factor reaches statistical significance ($p < 0.001$) but with a small effect size ($\eta^2_p = 0.031$), reflecting the slight detection range reduction at fat = 10 mm versus fat = 0 mm. Temperature effects and all interactions are non-significant ($p > 0.23$) with negligible effect sizes ($\eta^2_p < 0.003$). Because the 37 °C non-coaxial observations derive from batch B1 only, these results do not establish batch-independent thermal equivalence, and are interpreted as exploratory alongside the per-cell comparisons in §S16.3.

S16.3 Exploratory Temperature Comparisons

Table S18 compares available 22 °C and 37 °C detection distances in cells with successful measurements. For non-coaxial configurations, the 37 °C observations are from batch B1 only, whereas 22 °C summaries include three batches; these tests therefore confound temperature with batch structure and are reported as exploratory.

Table S18. Exploratory per-cell Welch comparisons of available 22 °C and 37 °C detection distances. Non-coaxial 37 °C data are limited to batch B1; p-values do not establish batch-independent thermal equivalence.

fat [mm]	θ_t	mean_22 ± SD	mean_37 ± SD	Δ [mm]	p_raw	p_Bonferroni	q_FDR	Significant?
0	0°	16.228 ± 0.276	16.168 ± 0.306	−0.060	0.166	0.996	0.332	no
0	30°	15.155 ± 0.392	15.237 ± 0.378	+0.082	0.313	1.000	0.375	no

0	60°	11.895 ± 0.509	11.789 ± 0.532	−0.106	0.343	1.000	0.375	no
0	90°	6.028 ± 0.397	6.004 ± 0.475	−0.024	0.816	1.000	0.816	no
10	0°	16.158 ± 0.301	16.065 ± 0.322	−0.093	0.048	0.288	0.288	no
10	30°	15.514 ± 0.157	15.536 ± 0.273	+0.022	0.849	1.000	0.849	no

No FDR-corrected comparison reached statistical significance in the available data. However, the absence of significance is not evidence of equivalence, and the unbalanced batch design does not support a general batch-independent thermal equivalence claim. Balanced multi-batch confirmation is required.

S17. Multi-Layer Phantom—Extended GUM Uncertainty Budget

This section reports the multi-layer phantom GUM budget using the standard uncertainty contributions listed in Table S20. Rectangular distribution source limits are converted to standard uncertainties before root-sum-of-squares propagation, giving $u_c = 0.640$ mm and expanded uncertainty $U(k=2) = \pm 1.28$ mm. The calculation remains conditional on the stated sensitivity coefficients and the limited three-batch estimate.

S17.1 Sensitivity Coefficients

Sensitivity coefficients are retained from the original model and are applied to source uncertainties before standardisation. Where a rectangular half-width is specified, the sensitivity-scaled half-width is divided by √3 before root-sum-of-squares propagation. Experimental repeatability terms are entered as standard deviations.

Table S19. *Sensitivity coefficients for principal uncertainty contributors.*

Contributor x_i	Type	Source uncertainty	Sensitivity c_i	Method
Linear stage position	B	±0.1 mm	1.0 (direct)	Manufacturer spec
Within-condition repeatability	A	s = 0.30 mm (n=30)	1.0	Experimental
Phantom ε_r tolerance	B	±10%	0.072 mm/% per layer	FEM finite-difference
Inter-batch variability	B	±0.45 mm (3 batches)	1.0	ANOVA between-group
Temperature ±0.5°C	B	±0.5°C	0.40 mm/°C	Empirical (37°C dataset)
RF/EMC noise	B	Variable per condition	0.150 mm	EMC test (§3.7)
Tag positioning depth	B	±0.2 mm	1.75 mm/mm depth	FEM finite-difference
Goniometer angular	B	±1° (random)	0.180 mm/°	Sin θ derivative

S17.2 Uncertainty Propagation

For rectangular source limits with sensitivity-scaled half-width a_i, the standard contribution is $u_i = a_i/\sqrt{3}$. For normally distributed repeatability terms, u_i is the estimated standard deviation. Table S20 reports the standard contributions used in the root-sum-of-squares calculation.

Table S20. *Uncertainty propagation: source values, distribution shapes, and standard uncertainty contributions.*

Contributor	Source value	Distribution	Divisor	u(x_i) [mm]	u_i [mm]
Linear stage	±0.1 mm half-width	Rectangular	√3	0.058	0.058
Repeatability	s = 0.330 mm	Normal	1	0.330	0.330
Phantom ε_r	±10% × 0.072	Rectangular	√3	0.416	0.416

Contributor	Source value	Distribution	Divisor	u(x_i) [mm]	u_i [mm]
	mm/%				
Inter-batch	s = 0.315 mm (3 batches)	Normal / limited-batch estimate	—	0.182	0.182
Temperature	±0.5°C × 0.40 mm/°C	Rectangular	√3	0.115	0.115
RF/EMC noise	s = 0.150 mm	Normal	1	0.150	0.150
Tag positioning	±0.2 mm × 1.75 mm/mm	Rectangular	√3	0.202	0.202
Goniometer	±1° × 0.180 mm/°	Rectangular	√3	0.104	0.104
Stage zero calibration	s = 0.060 mm	Normal	1	0.060	0.060

S17.3 Combined Standard and Expanded Uncertainty

The combined standard uncertainty is

$u_c = \sqrt{(0.058^2 + 0.330^2 + 0.416^2 + 0.182^2 + 0.115^2 + 0.150^2 + 0.202^2 + 0.104^2 + 0.060^2)}$

$u_c = \sqrt{0.4094} = 0.640$ mm

The expanded uncertainty at k = 2 is therefore

U = 2 × 0.640 = ±1.28 mm

The variance decomposition is dominated by phantom dielectric tolerance (42.3%), within-condition repeatability (26.6%), tag-positioning depth (10.0%), and inter-batch variability (8.1%). These percentages describe the present modelled measurement budget and should not be interpreted as clinical localisation accuracy.

The resulting expanded uncertainty of ±1.28 mm remains larger than the simpler bench-top budget and does not change the qualitative conclusion that phantom-specific uncertainty is greater. Further refinement requires the better characterisation of phantom dielectric sensitivity and more independent batches.

S17.4 Comparison with Saline-Medium GUM Budget

Table S21 compares the hybrid bench-top uncertainty estimate with the multi-layer phantom budget. The multi-layer standard uncertainty is approximately 2.1 times the bench-top value and the expanded uncertainty is approximately 2.0 times larger, reflecting additional phantom dielectric, batch, and positioning contributions.

Table S21. *Comparison of saline-medium and multi-layer phantom GUM uncertainty budgets.*

Quantity	Saline reference	Multi-layer phantom	Ratio
u_c (combined standard)	0.31 mm	0.640 mm	≈2.1×
U (expanded, k=2)	±0.63 mm	±1.28 mm	≈2.0×
Dominant contributor	Repeatability (49.0%) + inter-operator (32.7%)	Phantom dielectric (42.3%) + repeatability (26.6%)	shift
Number of contributors	7	9	+2
Number of measurements	480	2040	4.25×

The uncertainty budget is a metrological description of the phantom test process. It is not used to establish a clinical ±2 mm acceptance criterion, to claim 3D localisation accuracy, or by itself to assign a higher TRL.

S18. Supplementary Figures from Main Manuscript

This section provides system-level and validation figures supporting the main manuscript. Figures S1–S4 document the control architecture, sensor-board schematic, instrument mechanical layout, and bench-top test fixture. Figures S5–S7 summarise the hybrid bench-top uncertainty budget, multi-layer phantom dielectric characterisation, and descriptive comparison with the preceding bench-top antenna configuration.

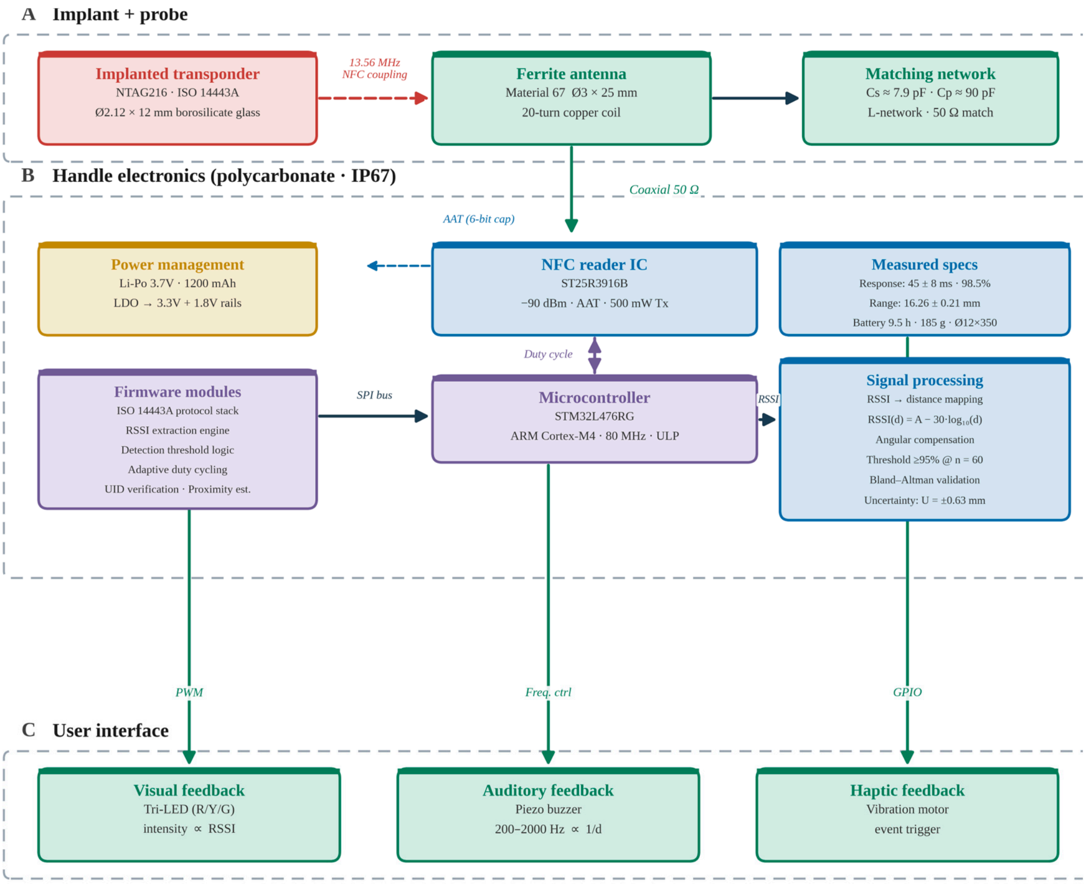

Figure S1. IntelliSense hierarchical control architecture. The primary detection loop provides the measured 45 ± 8 ms application-level response; power management implements state-dependent duty cycling; antenna tuning uses the ST25R3916B control outputs with external variable-capacitance elements; safety functions include UID verification, fault handling, and RF shutdown. Formal EMC and human-exposure compliance are outside the present study, and no continuous SAR-verification claim is made.

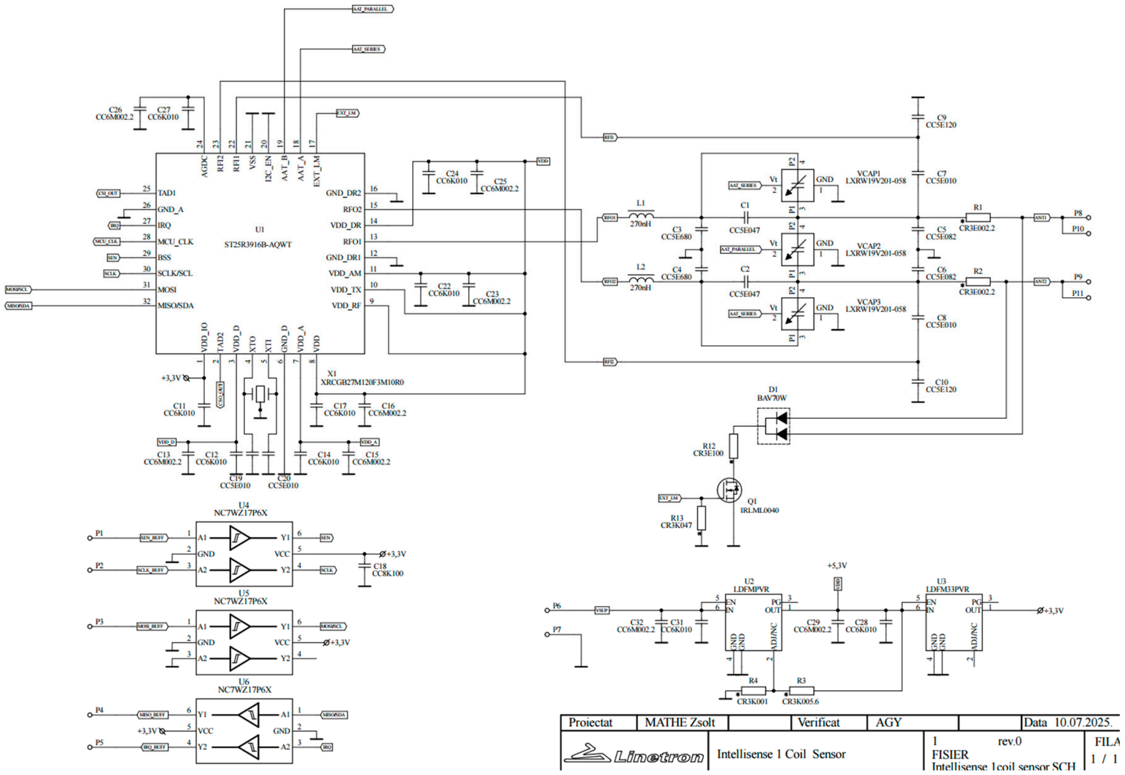

Figure S2. IntelliSense sensor board schematic. The ST25R3916B RF front end is connected to the ferrite-core antenna through the matching network. Automatic antenna tuning uses the reader AAT_A/AAT_B control outputs with external tuning diode/variable-capacitance elements. The 50 Ω reference applies to VNA characterisation, not to a fixed differential source impedance of the reader driver.

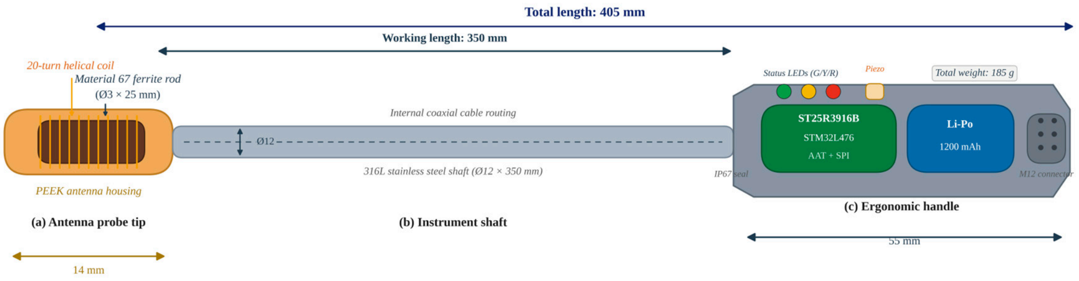

Figure S3. IntelliSense instrument schematic representation showing the antenna tip housing (ferrite-core), stainless steel shaft (Ø12 × 350 mm), coaxial cable routing, and ergonomic handle incorporating PCB, battery, and LED status indicators.

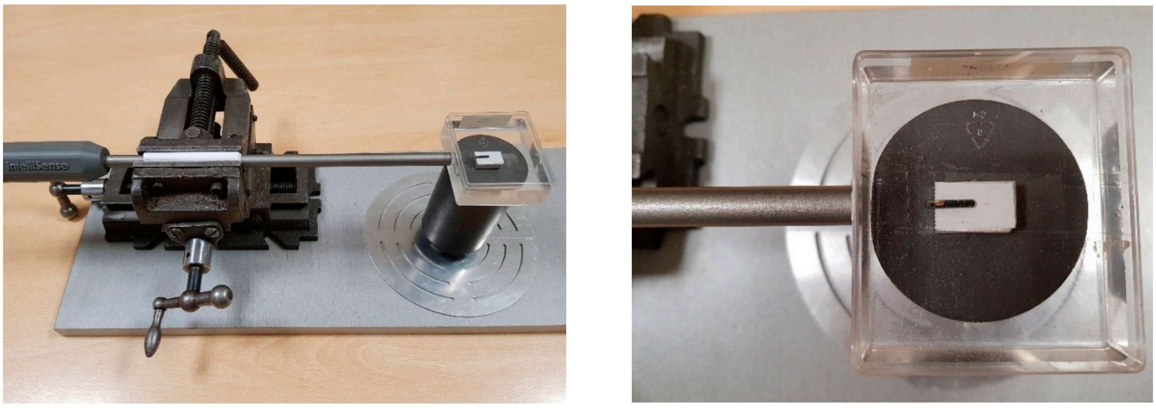

Figure S4. Bench-top test fixture for systematic IntelliSense characterisation. (a) Overall view of the fixture on the non-ferromagnetic aluminium baseplate, showing the two-axis cross-slide micrometric stage (X-axial, Y-lateral; resolution 0.1 mm) carrying the antenna assembly, the discrete spacer-based vertical positioning (Z; 0.5 mm increment), and the manual goniometer providing transponder rotation across 0°, 30°, 60°, 90°. (b) Detail of the PTFE sample-holder cup containing the NTAG216 transponder, fillable with air or 0.9% NaCl. The complete fixture is enclosed within a 60 dB RF-shielded measurement chamber (not shown).

Supplementary Figures S5–S7

Figure S5 visualises the hybrid bench-top GUM budget of main manuscript Table 8. The inter-operator term is a model-based pre-trial estimate rather than a directly quantified experimental component; accordingly, the figure represents a hybrid experimental/model-based uncertainty decomposition and should not be interpreted as a fully empirical reproducibility analysis.

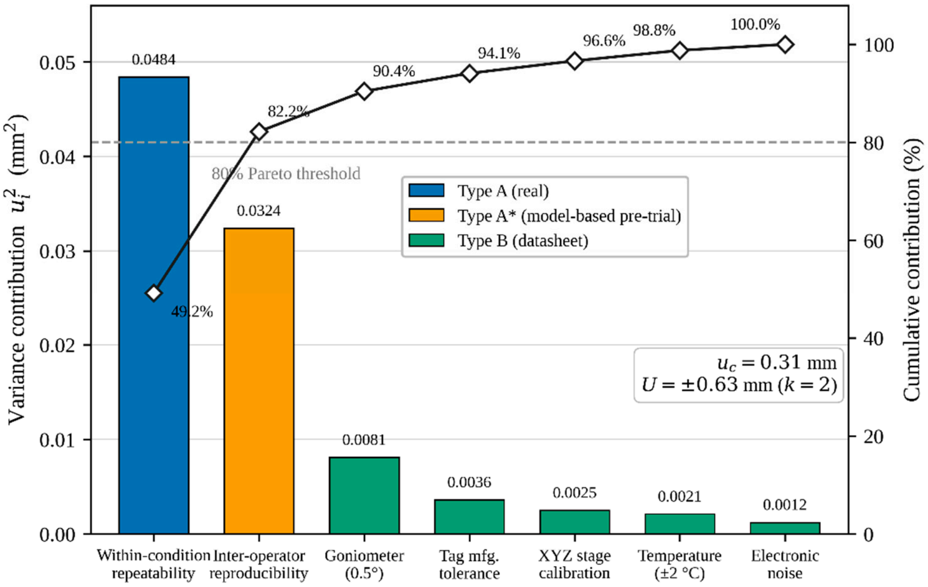

Figure S5. Hybrid bench-top uncertainty decomposition. Type A denotes measured within-prototype repeatability, A* denotes the model-based inter-operator term, and Type B denotes specification/model-derived contributions. Direct multi-operator validation remains pending.

Figure S6 summarises the multi-layer phantom dielectric characterisation of Table 9, comparing the measured permittivity (ε_r) and conductivity (σ) of each stratum at 13.56 MHz across three independent fabrication batches against the IT'IS Foundation 4.1 reference values; all strata remain within the ±10% compliance tolerance, evidencing batch-to-batch reproducibility.

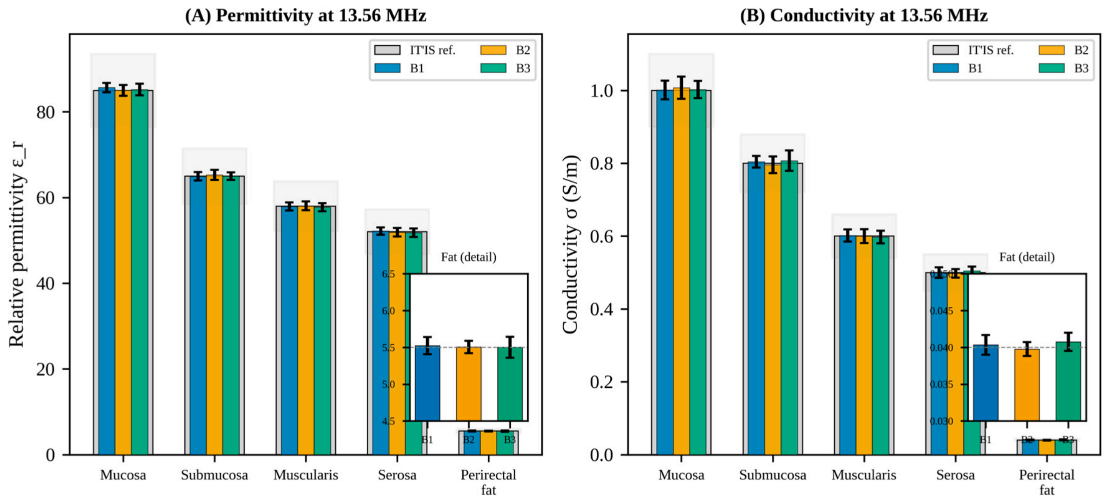

Figure S6. Multi-layer phantom dielectric characterisation across three batches. (A) Permittivity ε_r per stratum at 13.56 MHz. (B) Conductivity σ per stratum at 13.56 MHz. Three batches (B1, B2, B3) plotted against IT'IS Foundation 4.1 reference values (black bars) with ±10% tolerance bands (gray fills). All strata within compliance tolerance (n = 180 measurements per stratum).

Figure S7 provides descriptive benchmarking against the preceding Ø5 × 15 mm reference antenna. Geometry and detection-range comparisons are measured or directly derived, whereas unloaded-Q values are idealised/inferred estimates and are not used to claim measured Q-factor improvement.

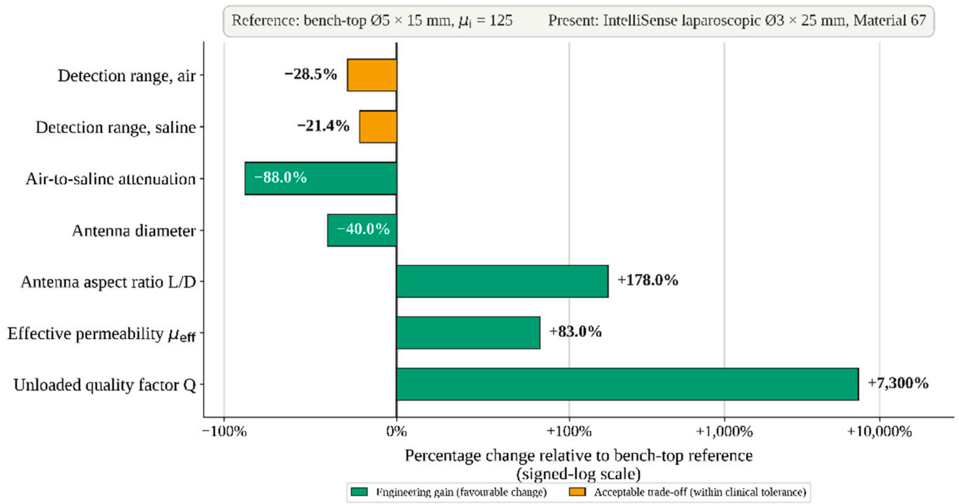

Figure S7. Descriptive comparison of the present Ø3 × 25 mm configuration with the preceding bench-top reference [21]. Detection range and geometry are the primary comparable metrics. Unloaded-Q percentage changes are not used because the analytical estimates are not equivalent to measured hardware gains.
